# Amyloid‐β but not tau accumulation is strongly associated with longitudinal cognitive decline

**DOI:** 10.1111/cns.14860

**Published:** 2024-07-16

**Authors:** Wenwen Wang, Jiani Huang, Shuangjie Qian, Yi Zheng, Xinyue Yu, Tao Jiang, Ruixue Ai, Jialong Hou, Enzi Ma, Jinlai Cai, Haijun He, XinShi Wang, Chenglong Xie

**Affiliations:** ^1^ The Center of Traditional Chinese Medicine, The Second Affiliated Hospital Yuying Children's Hospital of Wenzhou Medical University Wenzhou China; ^2^ Department of Neurology The First Affiliated Hospital of Wenzhou Medical University Wenzhou China; ^3^ Alberta Institute Wenzhou Medical University Wenzhou Zhejiang China; ^4^ Department of Clinical Molecular Biology, Akershus University Hospital University of Oslo Lørenskog Norway; ^5^ Department of Neurology Traditional Chinese and Western Medicine Hospital of Wenzhou Wenzhou Zhejiang China; ^6^ Oujiang Laboratory Wenzhou Zhejiang China; ^7^ Key Laboratory of Alzheimer's Disease of Zhejiang Province, Institute of Aging Wenzhou Medical University Wenzhou Zhejiang China; ^8^ Department of Geriatrics, Geriatric Medical Center The First Affiliated Hospital of Wenzhou Medical University Wenzhou Zhejiang China

**Keywords:** Alzheimer's disease, amyloid‐β, cognition function, tau accumulation

## Abstract

**Objective:**

Alzheimer's disease (AD) pathology is featured by the extracellular accumulation of amyloid‐β (Aβ) plaques and intracellular tau neurofibrillary tangles in the brain. We studied whether Aβ and tau accumulation are independently associated with future cognitive decline in the AD continuum.

**Methods:**

Data were acquired from the Alzheimer's Disease Neuroimaging Initiative (ADNI) public database. A total of 1272 participants were selected based on the availability of Aβ‐PET and CSF tau at baseline and of those 777 participants with follow‐up visits.

**Results:**

We found that Aβ‐PET and CSF tau pathology were related to cognitive decline across the AD clinical spectrum, both as potential predictors for dementia progression. Among them, Aβ‐PET (A + T− subjects) is an independent reliable predictor of longitudinal cognitive decline in terms of ADAS‐13, ADNI‐MEM, and MMSE scores rather than tau pathology (A − T+ subjects), indicating tau accumulation is not closely correlated with future cognitive impairment without being driven by Aβ deposition. Of note, a high percentage of APOE ε4 carriers with Aβ pathology (A+) develop poor memory and learning capacity. Interestingly, this condition is not recurrence in terms of the ADNI‐MEM domain when adding APOE ε4 status. Finally, the levels of Aβ‐PET SUVR related to glucose hypometabolism more strongly in subjects with A + T− than A − T+ both happen at baseline and longitudinal changes.

**Conclusions:**

In conclusion, Aβ‐PET alone without tau pathology (A + T−) measure is an independent reliable predictor of longitudinal cognitive decline but may nonetheless forecast different status of dementia progression. However, tau accumulation alone without Aβ pathology background (A − T+) was not enough to be an independent predictor of cognitive worsening.

## Graphical abstract

Aβ‐PET and CSF p‐tau181 were related to cognitive decline across the AD clinical spectrum, both as potential predictors for dementia progression. Aβ‐PET (A + T− subjects) was an independent reliable predictor of longitudinal cognitive decline rather than tau pathology (A − T+ subjects), indicating tau accumulation was not closely correlated with future cognitive impairment without being driven by Aβ deposition. Stronger predictive value of Aβ pathology (A+ alone) than tau accumulation (T+ alone) for longitudinal cognitive decline in APOE ε4 carriers.

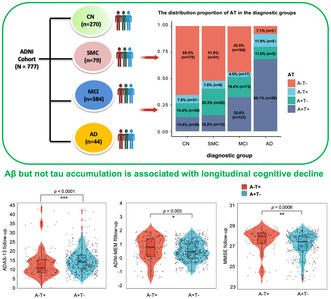

## 1 INTRODUCTION

Alzheimer's disease (AD) pathology is featured by extracellular accumulation of amyloid‐β (Aβ) plaques and intracellular tau neurofibrillary tangles in the brain, and no official approved regimes can change over or arrest the progression of this disease.^1^ Aβ deposition begins 15–20 years before clinical symptoms occur and precedes tau pathology in patients with AD.^2^ In the absence of Aβ, tau deposition might be insufficient to trigger the AD process. Because Aβ accumulation has been considered the initial attack that prompts either the tau pathology or tau‐mediated neurodegeneration in AD, and hence the development of AD medication has focused chiefly on removing Aβ from the brain.^3^ Nonetheless, numerous treatments that effectively reduce the Aβ load in the human brain have been exploited and carried out in clinical trials. Unfortunately, over the past 20 years, most present and past clinical trials of Aβ‐targeted therapeutics in sporadic late‐onset forms of AD have failed to show clinical benefits in terms of cognitive function despite reducing brain Aβ volume,^4^ as well as lacked suitable reflects of tau pathology.

Of note, regarding the spatiotemporal trajectory of both Aβ and tau deposition run through the whole brain, Aβ preferentially accumulates in high metabolic demand regions and spreads from the neocortex to the brainstem, eventually arriving at the cerebellum.^5^ Tau pathology, by comparison, first gathers obvious in the entorhinal cortex or locus coeruleus, from which then disseminates to limbic areas, and finally to the neocortex. Since Aβ and tau aggregations display distinct spatial and temporal patterns within vulnerable brain regions,^6^ this “spatial paradox” objects to the theory that tau accumulation is actuated by Aβ pathology happening in the same brain positions.^7^ Even then, tau pathology can also, albeit rarely, come up in defect of widespread Aβ plaque.^8^ In addition, apart from the topographical divergences, Aβ and tau also string along with different temporal sequences.^9^ The location of Aβ deposition is only mild‐modestly correlated with the brain neurodegeneration areas, and the degree of Aβ accumulation alleviates during advanced AD.^10^ By contrast to Aβ, either spatially or temporally, tau pathology correlates much more closely than Aβ pathology with cognitive impairment and neurodegeneration sites.^11^ In brief, the separate locations of tau and Aβ deposition and their natural progression in the cerebral represent that the two key biochemical pathologies progress relatively independently from each other.

Moreover, the other important matter to note is that there are several Aβ‐independent tau regulators in sporadic AD as already mentioned including cholesterol metabolism, endocytic system, APOE4, and activated microglia et al.,^12^ all act as upstream regulators of both Aβ and tau pathology, albeit through separate pathways. These findings indicate that Aβ‐targeting regimens alone might not be enough to halt the progression of tau pathology and cognitive decline in patients with AD. Thus, this study highlights uncertainty regarding the need to determine whether tau pathology is mainly driven by Aβ‐dependent or independent processes or whether Aβ and tau accumulation are independently associated with future cognitive decline in the AD continuum.

## 2 METHODS

### 2.1 Standard protocol approvals, registrations, and patients consent

All patients, or their partners, gave written informed consent to participate and all procedures bowed to ethical regulations for working with human subjects at each site, and the study was approved by the ADNI institutional review boards of all participating institutions and ethics committees.

### 2.2 Participants

All data in this study were obtained from the Alzheimer's Disease Neuroimaging Initiative (ADNI) database (http://adni.loni.usc.edu/), using ADNI‐1, ADNI‐GO, ADNI‐2, and ADNI‐3 studies. The ADNI is a continually forward observational project that was founded in 2003 as a public‐private partnership, hosted by Dr. Michael W. Weiner. Moreover, ADNI recruits subjects from 57 sites in the USA and Canada. Details regarding participants' inclusion and exclusion criteria and the full study protocol can be found at https://adni.loni.usc.edu/methods/documents/. We enrolled all ADNI participants (*N* = 1272) with at least one available baseline cerebrospinal fluid (CSF) p‐tau181 measurement, Aβ‐PET (^18^F‐Florbetapir‐PET or ^18^F‐Florbetaben‐PET), and cognition functions, including baseline and longitudinal cognitive assessments.

Moreover, demographic information entry (age, gender, education), genetic variables (APOE genotype), and Clinical diagnosis. Cognitive assessment scales mainly include the Mini‐Mental State Examination (MMSE), 13‐item Alzheimer's Disease Assessment Scale‐cognitive subscale (ADAS‐cog13), and Alzheimer's Disease Neuroimaging Initiative Memory Score (ADNI‐MEM). Moreover, 777 subjects had both Aβ‐PET and CSF tau information at baseline and follow‐up visits. All participants were divided into four groups according to clinical diagnosis, including cognitive normal function (CN: MMSE between 24 and 30, clinical dementia rating scale [CDR] = 0, memory box score = 0, nondepression), subjective cognitive impairment (SMC; MMSE between 24 and 30, CDR = 0, significant subjective memory problems reported by subjects, informants, CCI score ≥16), mild cognitive impairment (MCI: including early mild cognitive impairment [EMCI] or late mild cognitive impairment [LMCI]; MMSE between 24 and 30, CDR = 0.5, memory box score ≥0.5), and AD subjects (MMSE between 20 and 26, CDR ≥0.5, NINCDS/ADRDA criteria for probable AD). All the participant characteristics were presented in Table 1 and Table S1.

**TABLE 1:** Follow‐up participant characteristics of the ADNI participants (*N* = 777) according to clinical diagnosis.

Cohort, *n* (%)	CN	SMC	MCI	AD	*p* value
Group SUM	270 (34.7%)	79 (10.2%)	384 (49.4%)	44 (14.3%)	
ADNI 1	0	0 (0%)	7 (1.3%)	1 (2.3%)	
ADNI GO	0	0 (0%)	92 (24%)	0 (0%)	
ADNI‐2	139 (51.5%)	79 (100%)	235 (61.2%)	27 (61.4%)	
ADNI‐3	131 (48.5%)	0 (0%)	50 (13%)	16 (36.4%)	
Age (years)	71.5 (56.4,90.4)^d^	71.3 (60, 85.4)	71.2 (55.1,91.5)^d^	75.8 (55.6, 90.5)^a,c^	0.002
Weight (kg)	74.1 (63, 85.7)^c^	79.7 (65.9, 87.8)^d^	77.8 (69.1, 88.1)^a,d^	72.0 (62.9, 78.8)^b,c^	0.002
Sex, *n* (%)					
Male	116 (43%)	31 (39.2%)	214 (55.7%)	31 (70.5%)	
Female	154 (57%)	48 (60.8%)	170 (44.3%)	13 (29.5%)	
Education (years)	17 (16, 18)^c,d^	17 (16, 18)^d^	16 (14, 18)^a^	16 (14, 18)^a,b^	0.000
APOE ε4+/−	83 (30.7%) (*n* = 269)	28 (35.4%)	177 (46.1%) (*n* = 376)	29 (65.9%) (*n* = 43)	0.000
Cognition function					
MMSE	29 (29, 30)^c,d^	29 (29, 30)^c,d^	28 (27, 29)^a,b,d^	22 (21, 24)	0.000
ADAS13	8 (5, 11)^c,d^	8 (5, 12)^c,d^	14 (10, 18)^a,b,d^	29 (23, 35)^a,b,d^	0.000
ADNI_MEM	1.08 (0.69, 1.47)^c,d^	1.08 (0.84, 1.56)^c,d^	0.34 (−0.05, 0.82)^a,b,d^	−0.84 (−1.01, −0.49)^a,b,d^	0.000
Fluid biomarkers					
CSF Aβ42	1163 (847, 1589)^c,d^ (*n* = 237)	1058 (756, 1401)^c,d^ (*n* = 60)	832 (641, 1167)^a,b,d^ (*n* = 325)	616 (454, 768)^a,b,c^ (*n* = 42)	0.000
CSF Aβ40	18,510 (14,795,22,095) (*n* = 164)	NA	16,480 (14,170, 21,190) (*n* = 47)	16,210 (13,050,19,290) (*n* = 15)	0.380
CSF Aβ42/40	0.076 (0.05,0.1)^c,d^ (*n* = 164)	NA	0.048 (0.035, 0.085)^a^ (*n* = 100)	0.032 (0.029,0.045)^a^ (*n* = 34)	0.000
CSF p‐tau	19.2 (14.7, 24.7)^c,d^	20.7 (14.4, 26.3)^d^	21.8 (16.4, 32.0)^a,d^	33.6 (27.6, 46.7)^a,b,c^ (*n* = 43)	0.000
Tau+ (%)	57 (21.1%)	18 (22.8%)	142 (37%)	35 (79.5%)	0.000
CSF t‐Tau	211.9 (171.1, 276.3)^c,d^	220.1 (163.9, 300.1)^d^	239.9 (185.3, 325.0)^a,d^	335.9 (298.8, 440)^a,b,c^ (*n* = 43)	0.000
Plasma p‐tau181	13.4 (9.3, 19.0)^d^ (*n* = 139)	14.3 (10.5, 18.4)^d^	14.6 (10.6, 21.8)^d^ (*n* = 331)	25.0 (17.1, 29.0)^a,b,c^ (*n* = 27)	0.000
PET_SUVR, mean (SD)					
Amyloid_PET	1.03 (0.99,1,12)^b,c,d^	1.08 (1.01,1.24)^a,d^	1.14 (1.01,1.38)^a,d^ (*n* = 377)	1.47 (1.28,1.64)^a,b,c^ (*n* = 43)	0.000
Aβ + (%)	72 (26.7%)	32 (40.5%)	196 (51%) (*n* = 377)	35 (79.5%) (*n* = 43)	0.000

*Note*: Data are shown as P50 (P25, P75) or *n* (%). Continuous data were evaluated using Kruskal‐Wallis, categorical data were evaluated by Chi‐square test (χ^2^), and post hoc comparisons were adjusted by Bonferroni. Aβ positivity (A+) was defined as FBP and FBB SUVR exceeding 1.11 and 1.08, respectively; Tau positivity (T+) was defined as CSF p‐Tau >26.64 pg/mL.

Abbreviations: AD, Alzheimer disease; ADAS_13, 13‐item Alzheimer's Disease Assessment Scale‐cognitive subscale (ADAS‐cog13); ADNI, Alzheimer's Disease Neuroimaging Initiative; ADNI_MEM, Alzheimer's Disease Neuroimaging Initiative Memory Score; APOE−/+, noncarriers and carriers of the APOE ε4 allele; APOE, apolipoprotein E; Aβ42, β‐amyloid; CN, cognitively normal; CSF, cerebrospinal fluid; MCI, mild cognitive impairment; MMSE, Mini‐Mental State Examination; PET, positron emission tomography; p‐Tau, phosphorylated tau; SMC, subjective memory complaints; SUVR, standardized uptake value ratio (using a composite reference region) corrected for partial volume errors; t‐Tau, total tau. *p* values are given overall among CN, SMC, MCI, and AD. a: CN, b: SCD, c: MCI, d: AD *p* value<0.05.

### 2.3 Measurements of CSF and plasma biomarkers

ADNI Operator Manual describes the procedure for collecting CSF and plasma samples. CSF Aβ_42_, Aβ_40_, p‐tau, t‐tau (total tau), and plasma p‐tau181 available at baseline and the follow‐up visits during the study were included in this analysis. CSF Aβ_42_, Aβ_40_, p‐tau, t‐tau concentrations, and APOE status were obtained from the ADNI Biomarker Core Laboratory at the University of Pennsylvania Medical Center using fully automated Elecsys immunoassays whose timing matched Aβ‐PET data.^13^ The concentration of plasma p‐tau (pg/mL) was measured with a single‐molecule array (Simoa) HD‐1 analyzer (Quanterix) at the Clinical Neurochemistry Laboratory, University of Gothenburg (Molndal, Sweden), as described previously in detail.^14^ The cut‐off value for screening T+ using CSF tau was >26.64 pg/mL, a value previously published in a similar population suggests that this cut‐off represents the pathology of tau.^15^ Genotypes of APOE were determined using genetic testing for two single nucleotide polymorphisms (SNP; rs429358, rs7412). Genotypes were analyzed as dichotomous variables and the presence of at least one APOE ε4 genotype was considered APOE ε4 carrier.

### 2.4 PET imaging and imaging processing

The ADNI image acquisition methods can be found at: http://adni.loni.usc.edu/methods/pet‐analysis‐method/pet‐analysis/. In a nutshell, PET frames were realigned, averaged, reoriented, sliced into a common grid, and smoothed to a common resolution of 8 mm. A detailed description of PET acquisition and preprocessing can be found at www.adni.loni.usc.edu/methods/documents/. Details about the acquisition of the imaging measures have been reported elsewhere.^16^, ^17^ For quantitative PET analysis, in this experiment, we used Statistical Parametric Mapping 12 (SPM12) to register the pre‐processed PET images with their own PET and perform post‐smoothing modeling. Aβ positivity (A+) was defined as FBP and FBB SUVR exceeding 1.11 and 1.08, respectively.

### 2.5 Cognitive assessments

We downloaded cognitive scales at baseline and yearly follow‐up, including ADNI‐MEM, a combination of the Rey Auditory Verbal Learning Test, Alzheimer's Disease Assessment Scale, Wechsler Logic Memory Scale I and II, and MMSE word recall, ADAS‐cog13, and MMSE as tools to track cognitive measurement trajectories. We recorded the perceptions available each year during the follow‐up of included participants and calculated the annual rate of cognitive change from the previous year as a baseline, with a mean follow‐up time of 4.76 years. For the MMSE or other cognitive scales, all included participants will be assessed at the time of enrollment. In the ADNI database, and then follow‐up every year thereafter. Indeed, during the follow‐up process of MMSE, participants will have an impact on the results due to learning effects. Therefore, for cognitive function assessment, we need to combine multiple cognitive scale assessments to make comprehensive judgments, such as ADAS‐13, ADNI‐MEM, and MMSE together. Moreover, we superimposed the time of follow‐up and the number of people followed per year and estimated follow‐up cognition using a linear mixed model with baseline cognition and sex, age, education, and APOE as fixed factors and follow‐up time as a random factor, to deduce the cognitive changes of MMSE, ADAS‐13, and ADNI‐MEM.

### 2.6 Statistical analysis

The differences in baseline characteristics between diagnostic groups were evaluated using Kruskal–Wallis for continuous data, chi‐squared tests (χ^2^) for categorical data, and Bonferroni‐adjusted post hoc comparisons. Distributions of AT in the differential diagnosis have been visualized by hierarchical histograms and Sankey diagrams. The correlation of cognitive assessments (baseline and follow‐up) and biomarkers (Aβ‐PET and CSF tau) was assessed using the Spearman rho correlation coefficient (Rho) (*p* < 0.05, unadjusted for multiple comparisons). Statistical significance was assumed at *p* ≤ 0.05 (two‐sided).

### 2.7 Linear and mixed‐effects models predicted baseline and longitudinal cognition

Afterward, Linear models controlling for age, sex, education, and APOE were used to estimate baseline cognition (ADAS‐13, ADNI‐MEM, and MMSE) across the different AT groups; To further assess the role of biomarkers status (Aβ‐PET and CSF tau) in predicting a longitudinal cognitive decline, we performed a linear mixed‐effects models (LMM) to estimate follow‐up cognition (controlling for age, sex, education, APOE, and baseline cognition as fixed factors and controlling for follow‐up time as a random factor), where we included a superposition of annual follow‐up cognition and the number of eligible participants. Regarding the model estimation, the models with different combinations of variables were tested, the most appropriate model for the Akaike Information Criterion (AIC) and the Bayesian Information Criterion (BIC) was selected, and LMM significance tests were performed using *p* ≤ 0.05. We calculated standardized regression coefficients *β* for different AT groups to compare the effect of different AT groups on baseline and follow‐up cognition in the absolute magnitude of *β*. To further explore the independent role of Aβ‐PET and CSF tau accumulation in cognitive change, we again performed regression analysis using a mixed‐effects model with follow‐up cognition as the dependent variable and CSF tau or Aβ‐PET as the predictor variable, stratified by Aβ or tau status. In addition, to further obtain the independent effects of Aβ and tau on cognition, we selected participants who were always A + T– (*N* = 115) and A − T+ (*N* = 40) before and after follow‐up as a subsample to repeat the above statistical analysis with the same methods described above. Besides, to understand the role of the APOE ε4 allele, stratified analysis was performed to estimate the APOE genotype in the above model, and we examined the interaction between AT and APOE ε4 status in longitudinal follow‐up cognition in A − T+ and A + T− classification subsamples. For the LMM analysis, we used the “lmer Test” and “lme4” R packages.

### 2.8 Voxel correlation analysis

First, we performed a linear voxel‐wise correlation analysis of baseline Aβ‐PET and baseline cognition (ADAS‐13, ADNI‐MEM, and MMSE scales) controlling for age, sex, education, and APOE from the cross‐sectional profile, and then again performed similar model estimates for tau (+) and tau (−) subgroups. In addition, to compare the independent prospective effects of Aβ and tau pathology on cross‐sectional and longitudinal glucose hypometabolism, we performed two‐sample t‐tests of FDG‐PET levels at baseline (*N* = 131) and 2‐year follow‐up (*N* = 75) in the A − T+ and A + T subgroups, and multivariate linear voxel‐wise correlation analyses of FDG‐PET with Aβ‐PET and CSF tau pathology in cross‐sectional and longitudinal directions. Neuroimaging analysis was performed using the toolkit SPM12 (multiple regression, two‐sample t‐test) in MATLAB 2020A (MathWorks, Inc.). Results from voxel‐wise analyses were assessed using family‐wise error‐corrected significance thresholds (PFWE <0.05) at the cluster level with an initial voxel‐wise height threshold of *p* < 0.05 and a projection of results from voxel‐wise analyses using the BrainNet Viewer toolkit. All statistical analyses were performed using R (version 4.2.1).

## 3 RESULTS

### 3.1 Participants in the ADNI database

**FIGURE 1:**
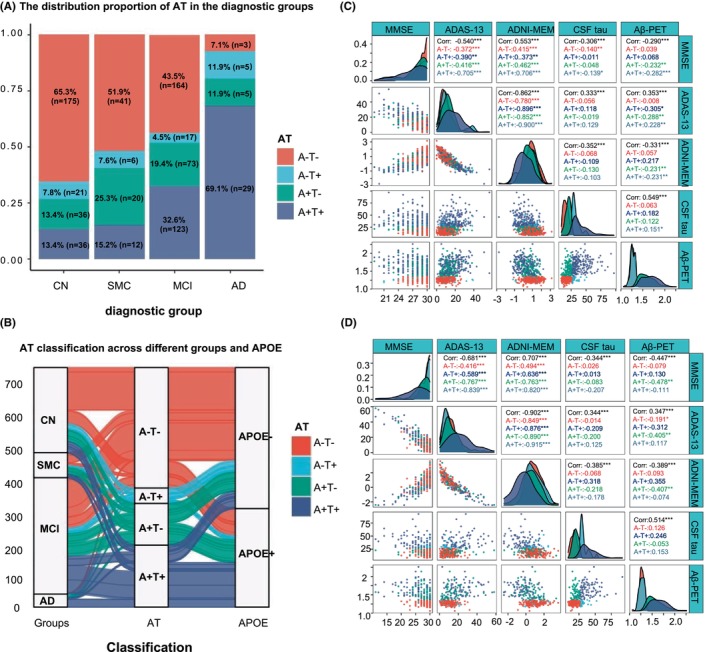
Associations of Amyloid‐β and tau accumulation with the cognitive decline across the AD clinical spectrum. (A, B) Distribution of AT in the differential diagnosis and AT groups; (C, D) Multivariate correlation plots show Pearson correlations for baseline and follow‐up cognition tests (MMSE, ADAS‐13, and ADNI‐MEM) and AT biomarkers (amyloid‐PET and CSF tau) in different AT subgroups, respectively. AD, Alzheimer's disease; CN, cognitively normal; MCI, mild cognitive impairment; SMC, subjective memory concern.

Detailed baseline participant demographic data were summarized in Table S1. A total of 1272 participants were selected, comprising 364 individuals with normal cognition (CN), 96 clinically diagnosed with SMC, 544 individuals with MCI, and 168 Alzheimer's disease patients. The distribution proportion of AT classification across the diagnostic groups is in Figure 1A,B. We found the sample size of A − T− people (*n* = 3) in the AD group is too low, which reflects the accuracy of this clinical diagnosis. In the ATN system, the Aβ status (A+) is essential to consider an individual in the AD continuum due to it reflects AD‐related brain pathological changes in the preclinical or earliest phases of the disease. By contrast, negativity in the A domain (A−) points that the individual does not have AD‐like pathology and is unlikely to be on the AD continuum.^18^ The mean age of patients with CN, SMC, and MCI was significantly lower than that of AD participants. There were more female patients with CN and SMC (59.3% of CN and 59.4% of SMC) compared to MCI and AD (44.9% of MCI and 38.7% of AD) and low education levels in the MCI and AD groups. Participants carrying the APOE ε4 allele were particularly overrepresented in AD and MCI groups (66.7% and 46.7%, respectively), as expected, compared with SMC (33.3%) and CN (30%). As indicated by ADAS‐13, ADNI‐MEM, and MMSE (Mini‐Mental State Examination) cognitive function scores were significantly different between AD and other groups. To characterize individuals with Aβ and tau abnormality indicated by CSF assay or PET imaging. Data‐driven, clinically relevant thresholds for CSF and neuroimaging Aβ and p‐tau data were also incorporated in Table S1. There was a downward trend of lower CSF Aβ_42_, and Aβ_42/40_ in the AD compared to other groups. Of interest, there were no differences in CSF Aβ_40_ levels in each group. Conversely, the levels of p‐tau and t‐tau in CSF were significantly higher in the AD group. The distribution of “T+” based on the CSF p‐tau cut‐off value was 69.6% in AD, namely, close to 30% was sparse from tau pathology. In terms of Aβ‐PET neuroimaging, the SUVR was dramatically increased in the AD group compared to the other three groups. 85.7% of participants were located to “A+” in AD according to the SUVR cut‐off value, and 14.3% were free of Aβ pathology, indicating Aβ and tau were partially independently associated with the AD diagnostic group.

### 3.2 Associations of Aβ and tau accumulation with the cognitive decline across the AD clinical spectrum

The linear correlation at baseline between cognitive scores and CSF p‐tau level was similar in value to the correlation between cognitive scores and neuroimaging Aβ‐PET (Figure 1C). First, we tested whether Aβ‐PET (A element) was associated with worsened cognitive performances, including ADAS‐13, ADNI‐MEM, and MMSE. As anticipated, Aβ‐PET was highly correlated with the primary outcome ADAS‐13 (*r* = 0.353, *p* < 0.001, Figure 1C), and also for the secondary outcomes ADNI‐MEM (*r* = −0.331, *p* < 0.001, Figure 1C) and MMSE (*r* = −0.290, *p* < 0.001, Figure 1C) in AD spectrum participants. Regarding tau pathology (T element), memory performance was also closely associated with the CSF p‐tau levels (ADAS‐13: *r* = 0.333, *p* < 0.001, ADNI‐MEM: *r* = −0.352, *p* < 0.001, and MMSE: *r* = −0.360, *p* < 0.001, respectively, Figure 1C). Afterward, we explored whether Aβ‐PET and CSF p‐tau were related to faster cognitive change during the follow‐up visits (4 years) using the linear correlation analysis. Meanwhile, follow‐up participants' basic demographic data and biomarkers values were summarized in Table 1 and Table S2. Significant Aβ‐PET‐related differences in longitudinal ADAS‐13 (*r* = 0.347, *p* < 0.001, Figure 1D), ADNI‐MEM (*r* = −0.389, *p* < 0.001, Figure 1D), and MMSE (*r* = −0.447, *p* < 0.001, Figure 1D) changes were detected in this cohort. Similarly, correlation analysis showed that annual cognitive change rates were associated with tau accumulation in terms of CSF p‐tau levels in all cognitive tests (ADAS‐13: *r* = 0.344, *p* < 0.001, ADNI‐MEM: *r* = −0.385, *p* < 0.001, and MMSE: *r* = −0.344, *p* < 0.001, respectively, Figure 1D).

### 3.3 Aβ deposition is an independent reliable predictor of cognitive decline compared to tau pathology

**FIGURE 2:**
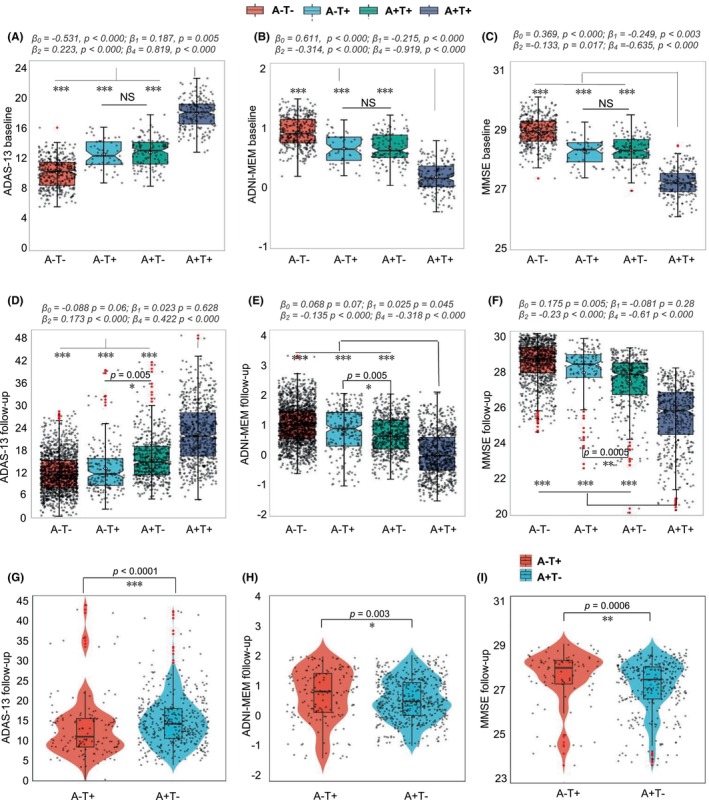
Amyloid‐PET is an independent reliable predictor of cognitive decline rather than tau pathology. (A–C) Box and whisker plots illustrate baseline cognition estimated after the linear regression model (controlling for age, sex, education, and APOE status) across the different AT groups; (D–F) Box and whisker plots illustrate follow‐up cognition estimated after the linear mixed model (controlling for age, sex, education, APOE baseline cognition as a fixed factor and controlling for follow‐up time as a random factor) across the different AT groups; (G–I) Violin plots illustrate follow‐up cognition estimated after the linear mixed model across the subgroups that were consistently A − T+ and A + T− before and after follow‐up. Standardized *β*‐ and *p*‐values were derived from linear regression. The central black lines show the median value, upper and lower quartiles, respectively, display the upper and lower quartiles of the black line; the white diamond represents the mean of cognition; the red points are outliers. The number of follow‐up cognitions was superimposed on the number of repeated measures and the number of follow‐up visits. *p ≤ 0.01, **p ≤ 0.001. ***p ≤ 0.0001. Concrete model estimates are shown in Table S6.

For the trajectory of longitudinal memory performances and cognition conversion rate in different AT groups, we observed the annual memory scales change tendency at the follow‐up visits versus baseline (Figure S1; Table S3). To further address whether tau pathology can progress independently of Aβ deposition and to be an independently reliable predictor of cognitive decline in the AD continuum and vice versa. The results of the linear regression model analysis after adjusting age, education, and sex, showed a significant A + T+ effect on ADAS‐13 (Figure 2A), ADNI‐MEM (Figure 2B), and MMSE levels (Figure 2C). Regarding the groups between A − T+ and A + T− as the core comparisons, we found no significant difference in cognitive levels at baseline (Figure 2A–C), indicating T+ and A+ both independently influence cognitive function in a similar degree at baseline. The corresponding statistical analysis values can be found in Table S4. We next performed linear mixed‐effect models (LMM) analysis to test the effects of A+ and T+ alone on longitudinal cognition changes, as a potential longitudinal predicting factor. LMM analysis results showed an analogical tendency compared with the linear regression model, namely, there was an apparent interaction between A + T+ classification on ADAS‐13, ADNI‐MEM, and MMSE levels change (Figure 2D–F; Table S4). However, there were nearly no associations between changes in ADAS‐13 (*β1* = 0.023, *p* = 0.628, Figure 2D) and MMSE (*β1* = −0.081, *p* = 0.28, Figure 2F) over time and A − T+ classification, suggesting T+ alone sparing with A+ background was not enough independent predictor of cognitive decline. Of note, worsened cognition performance in the A + T− was observed when compared to A − T+, indicating A+ displayed a close relationship with longitudinal cognitive decline. The detailed temporal trajectories of memory test data and annual cognitive change rates were displayed in Tables S3 and S5.

**FIGURE 3:**
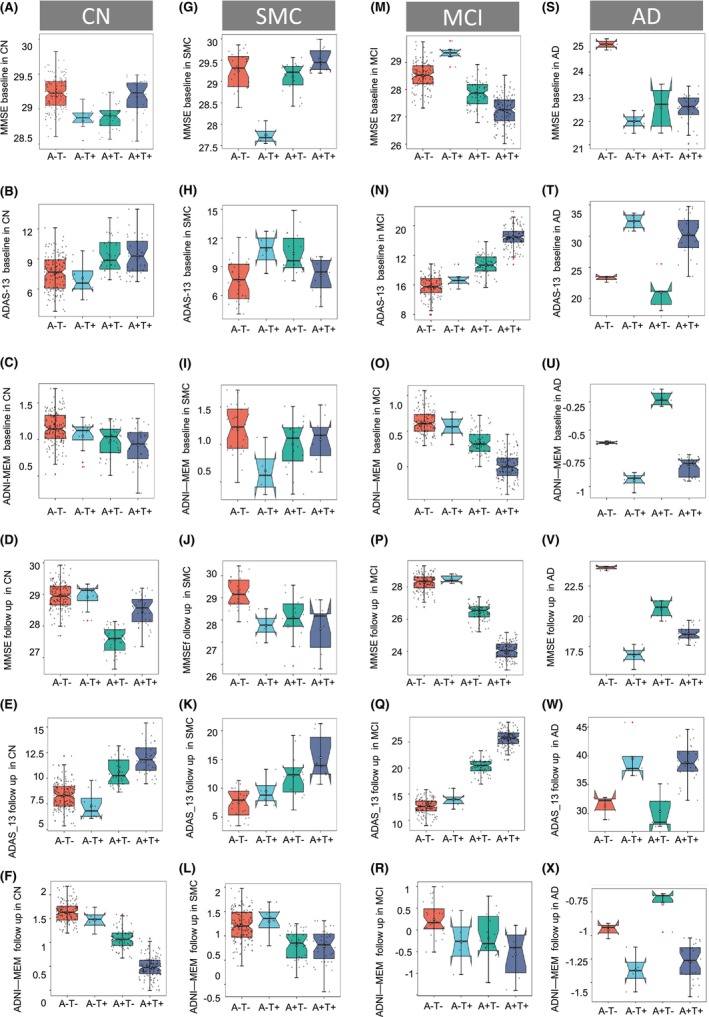
The cognition discrepancy between A and T inside AD/MCI/SMC/NC groups. Box and whisker plots illustrate baseline cognition estimated after the linear regression model (controlling for age, sex, education, and APOE status) across the different AT groups in CN (A–F), SMC (G–L), MCI (M–R), and AD (S–X). The number of follow‐up cognitions was superimposed on the number of repeated measures and the number of follow‐up visits. The detailed information can be found in Table S7.

Further, analyses stratified by Aβ status demonstrated that CSF tau is associated with a mild influence on the longitudinal ADAS‐13 (Figure S2a), ADNI‐MEM (Figure S2b), and MMSE (Figure S2c) changes in **Aβ**‐negative subjects (A + T−). As expected, the effect showed greater when under the **Aβ**‐positive subjects (A + T+). By comparison, Aβ‐PET SUVR could predict dementia progression no matter whether CSF tau was positive or negative (Figure S2d–f), suggesting **Aβ**‐PET was an independently reliable predictor of cognitive decline in the AD continuum (Table S6). To exclude the risk of A (+/−) or T (+/−) components change over the follow‐up visits, we further select the subjects keeping A + T− or A − T+ before and after follow‐up and analyzed the independent predicting dementia progression value. We found that the pattern of A + T− and A − T+ correlation with performance on memory and executive functioning tests across participant groups was consistent with the aforementioned analysis's results. Dementia progression over time was highly correlated with A + T− classification compared to A − T+ modality (ADAS‐13, *p* < 0.0001; ADNI‐MEM, *p* = 0.0032; MMSE, *p* = 0.0006, Figure 2G–I), indicating Aβ‐PET was indeed an independent reliable predictor of cognitive decline when comparing to tau pathology. Moreover, we only adopted Aβ‐PET and CSF tau to reflect the A and T components due to the sample consideration in this study. Therefore, to investigate whether the changes in PET were highly linked to CSF or vice versa. A linear correlation analysis model displayed that CSF Aβ_42/40_ or CSF tau variations were sensitive to Aβ‐PET or tau‐PET (Figure S2g,h), implying the close concordance between PET and CSF, which was in line with some previous papers.^15^, ^19^ We also tested the difference between A and T inside AD/MCI/SMC/NC groups for the statistic comparison in terms of the cognitive levels at baseline and follow‐up visits. The detailed information can be found in Figure 3 and Table S7.

**FIGURE 4:**
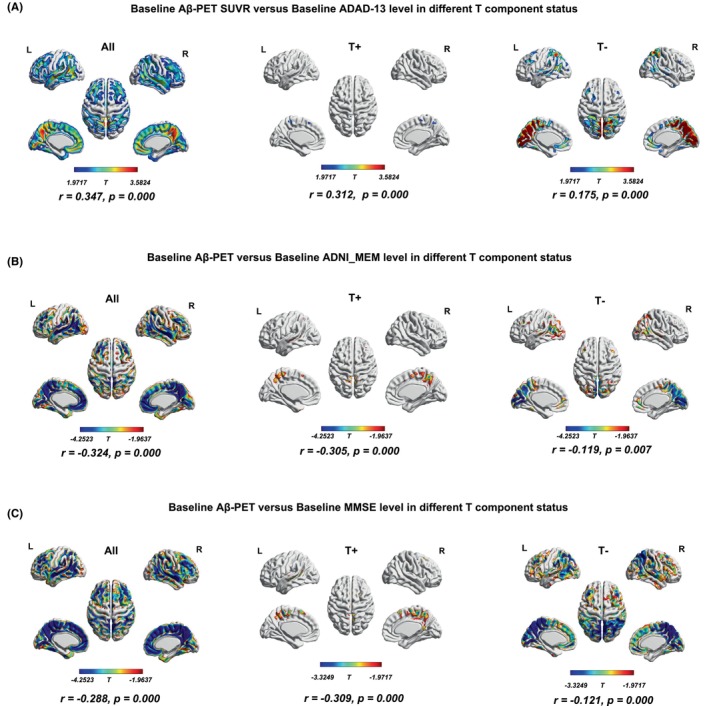
Cross‐sectional associations of baseline Aβ‐PET SUVR versus baseline cognitive levels in different T component status using voxel‐wise analyses. Cognition on the baseline ADAS‐13 (A), ADNI‐MEM (B), and MMSE (C) scales and voxel multivariate regression analysis of amyloid (controlling for age, sex, education, and APOE), respectively, voxel regression was again performed in the Tau+ and Tau− subgroups in the total sample; we did a partial correlation analysis of amyloid‐PET and three baseline cognition items, respectively (adjusted for age, sex, education, and APOE). *r* was the partial correlation coefficient. ADAS‐13‐related brain regions, the deeper the red color, the greater the absolute value of T, the stronger the correlation; MMSE or ADNI‐MEM‐related brain regions, the deeper the blue color, the greater absolute value of T, the stronger the correlation. FWE, family‐wise error corrected.

Next, we assessed the cross‐sectional associations of baseline Aβ‐PET SUVR versus ADAD‐13 levels in different T component status using voxel‐wise analyses (adjusted for age, sex, education, and APOE4 status). Baseline levels of Aβ‐PET SUVR related with ADAS‐13 more strongly in subjects with tau positive subjects (*r* = 0.312, *p* < 0.0001), while the association was still significant difference among tau negative participants (*r* = 0.175, *p* < 0.0001, Figure 4A). We then investigated the correlations of baseline Aβ‐PET SUVR versus ADNI‐MEM and MMSE and also found the apparent correlation both in tau positive and negative subjects (Figure 4B,C). Inevitably, the results of linear regression analysis showed a significant Aβ‐PET × tau accumulation interaction effect on memory test levels.

### 3.4 Stronger predictive capability of Aβ pathology for cognitive decline in APOE ε4 carriers compared to tau accumulation

**FIGURE 5:**
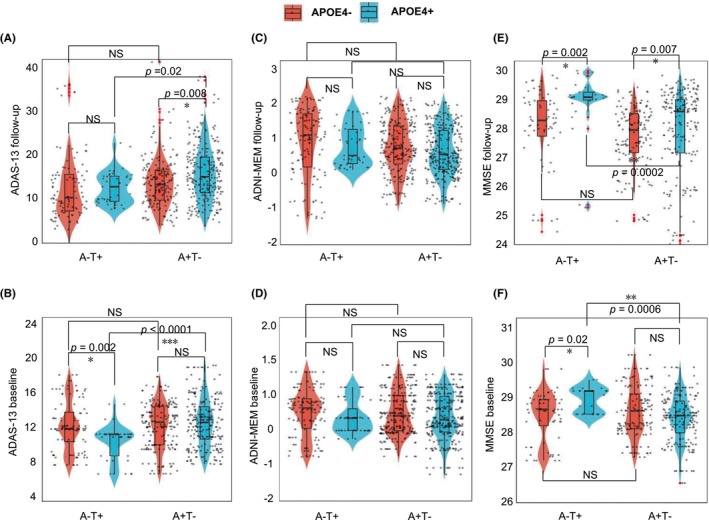
Stronger predictive value of Aβ pathology for cognitive decline in APOE ε4 carriers compared to tau accumulation. Violin plots illustrate the distribution of following‐up cognition scores including ADAS‐13 (A, B), ADNI‐MEM (C, D), and MMSE (E, F) estimated after the linear mixed model (controlling for age, sex, education, baseline cognition as a fixed factor and controlling for follow‐up time as a random factor) across the different AT groups (A + T− and A − T+) and APOE ε4 groups; The central black lines show the median value, upper and lower quartiles, respectively, display the upper and lower quartiles of the black line; the white diamond represents the mean of cognition; the red points are outliers. The weight of the violin represents the density of the population distribution; the AT subgroups were A−T+ and A + T− subgroups that remained unchanged before and after follow‐up. *p ≤ 0.01, **p ≤ 0.001. ***p ≤ 0.0001. Concrete model estimates are shown in Table S5.

A linear mixed‐effects model (with random intercept) of memory scores over 4 years was used to analyze the predictive properties of Aβ pathology (A + T−) and tau accumulation (A − T+) with or without APOE ε4. Regarding the ADAS‐13 domain, we found that carriers of the APOE ε4 (APOE+) allele had abnormally worsened longitudinal cognition performance both in A + T− and A − T+ classifications (Figure 5A), especially these abnormalities were observed in A + T− when compared to the A − T+ groups, who were free of Aβ pathology (*p* = 0.02, Figure 4A; *p* < 0.0001, Figure 5B). Of note, in line with ADAS‐13, APOE ε4 carriers developed a lower value of MMSE (*p* = 0002, *p* = 0.0006, respectively, Figure 4E,F). These results suggested that a high percentage of APOE ε4 carriers with Aβ pathology (A+) develop poor future memory and learning capacity. Interestingly, this condition was not recurrence in terms of the ADNI‐MEM domain when adding APOE ε4 status (Figure 5C,D; Table S7). Moreover, we want to check to see what happens with other microglia‐related genes in those patients with different levels of A, T, and cognitive functions. A previous study suggested that the BIN1 rs744373 SNP is associated with increased tau but not Aβ pathology, indicating that alterations in BIN1 may contribute to memory deficits via increased tau pathology.^20^ CD33 is a transmembrane protein that inhibits microglial clearance of Aβ.^21^ We included CD33 as microglia alleles in this issue and found that carriers of the CD33 risk allele had no difference in cognition performance (ADAS‐12 and ADNI‐MEM) at baseline and longitudinal between A + T− and A − T+ classifications (Figure S3).

### 3.5 Aβ accumulation is an independent predictor of cross‐sectional and longitudinal glucose hypometabolism

**FIGURE 6:**
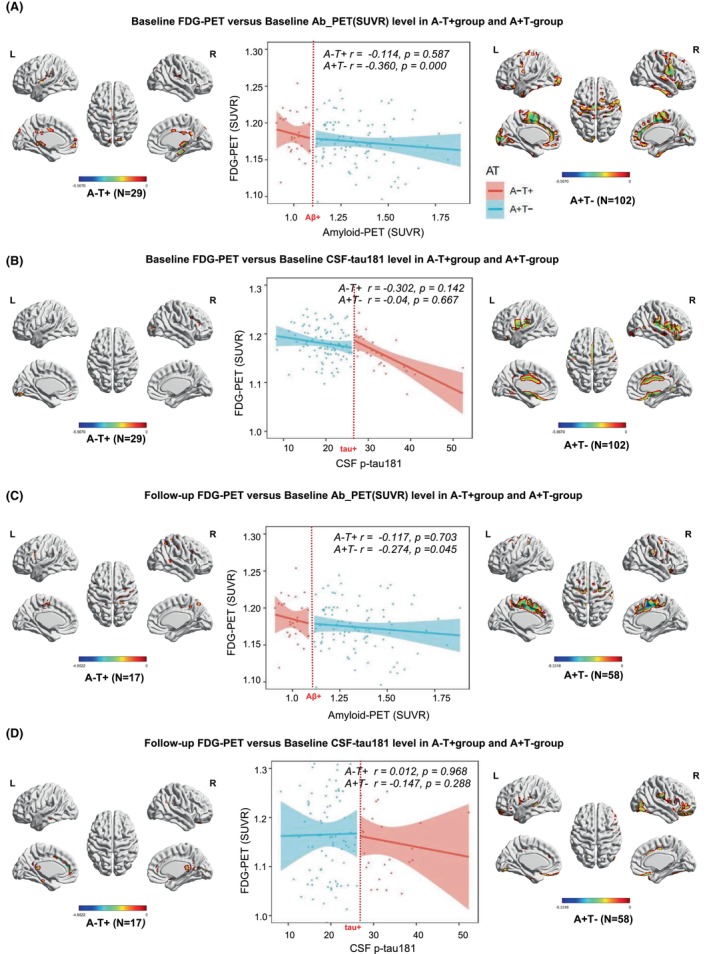
Amyloid‐PET is an independent predictor of cross‐sectional and longitudinal glucose hypometabolism. (A) Correlation between baseline FDG‐PET and baseline Aβ‐PET SUVR levels in A − T+ and A + T− groups; Scatterplot showing the correlations between baseline FDG‐PET SUVR and baseline Aβ‐PET SUVR levels; (B) Correlation between baseline FDG‐PET and baseline CSF tau levels in A − T+ and A + T− groups; Scatterplot showing the correlations between baseline FDG‐PET SUVR and baseline CSF tau levels; (C) Correlation of baseline Aβ‐PET versus longitudinal FDG‐PET SUVR change; (D) Correlation of baseline CSF tau versus longitudinal FDG‐PET SUVR change.

In terms of the neurodegeneration domain, especially in the form of glucose hypometabolism (FDG‐PET), appears to be vital for deteriorating cognitive decline and furthering its progression to the AD stage. First, we assessed the cross‐sectional associations of Aβ‐PET and CSF tau with FDG‐PET SUVR across the AD continuum using voxel‐wise analyses (adjusted for age, sex, and APOE4). Baseline levels of Aβ‐PET related with glucose hypometabolism more strongly in subjects with A + T− (*r* = −0.360, *p* = 0.000) than A − T+ (*r* = −0.114, *p* = 0.587, Figure 6A), while the association was markedly weaker among CSF tau and FDG‐PET SUVR (A − T+: *r* = −0.302, *p* = 0.142; A + T−: *r* = −0.04, *p* = 0.667, Figure 6B). Moreover, the close associations found in A + T− participants were restricted to the Brodmann Area 4 (Primary Motor), 20 (Inferior Temporal gyrus), 32 (Dorsal anterior cingulate cortex), and 43 (Subcentral area) areas. We then investigated the correlations of baseline Aβ‐PET versus longitudinal FDG‐PET SUVR change and found a significant correlation also in A + T− group (*r* = −0.274, *p* = 0.045, Figure 6C) compared to A − T+ subjects (*r* = −0.117, *p* = 0.703, Figure 6C). Remarkably, voxel‐wise analyses investigated the relationships between CSF tau and FDG‐PET change. Similar to baseline results, no significant association was found both in A − T+ (*r* = 0.012, *p* = 0.968, Figure 6D) and A + T− groups (*r* = −0.147, *p* = 0.288, Figure 6D). Of note, we found that Brodmann 2 (Somatosensory Cortex), 4 (Primary Motor Cortex), 24 (Ventral Anterior cingulate cortex), and 32 (Dorsal anterior cingulate cortex) brain regions FDG‐PET SUVR longitudinal change were significantly closely correlated with Aβ‐PET levels. Meanwhile, various patterns and region‐specific effects of Aβ (A + T−) and tau pathology (A − T+) on baseline FDG‐PET SUVR and longitudinal change were shown in Figure S4.

## 4 DISCUSSION

The three core findings of the largest prospective longitudinal ADNI study thus far were as follows^1^: Aβ‐PET and CSF tau pathology were related to the cognitive decline across the AD clinical spectrum, both as potential predictors for dementia progression.^2^ Aβ‐PET (A + T− subjects) was an independent reliable predictor of longitudinal cognitive decline compared to tau pathology (A − T+ subjects), indicating tau accumulation was not closely correlated with future cognitive impairment without being driven by Aβ deposition. Aβ and tau had different roles in predicting clinical dementia status across the disease progression.^3^ Stronger predictive value of Aβ pathology (A+ alone) than tau accumulation (T+ alone) for longitudinal cognitive decline in APOE ε4 carriers. Taken together, these data showed that Aβ could accurately predict future disease progression and contribute to dementia even if without following tau pathology, while tau needs to be accompanied by Aβ‐driven together. Given that AD histopathology accrued for several decades before the clinically apparent cognitive impairment,^2^ it should be urgent to screen asymptomatic or mild memory complaint adults who are likely to develop AD dementia in the future. Consistent with previous results,^22^, ^23^ we found that Aβ and tau pathology both were related to low cognition performance across the AD clinical spectrum.

Genetic data, neuroimaging, and even autopsy studies in AD continuum patients indicate that Aβ deposition precedes tau pathology.^24^ Due to Aβ accumulation has been supposed the earliest insult that actuates the accumulation of tau in AD. The tau‐PET signal does not consistently increase over time in cognitively normal individuals with Aβ‐negative (+), whereas deposition of tau in the cortexes increases by 0.5–3.0% annually in cognitively normal persons who are Aβ (+), and by 3.0–8.0% in Aβ‐positive patients with clinical AD.^25^, ^26^ In addition, serious tau pathology in the cortex is more often perceived in Aβ (+) cognitively normal persons than in Aβ (−) cognitively normal individuals.^27^ These findings have shed light on the theory that AD is an Aβ‐promoted tauopathy in which the presence of Aβ ignites the spread of tau. However, by contrast, emerging preclinical work is inconsistent with this idea, and instead vindicates the concept that several biological pathways involved in sporadic AD, drive tau accumulation in an Aβ‐independent manner.^12^ Of note, in the neuropathology studies, the finding of tau tangles in the basal forebrain, medial temporal lobe, and olfactory areas in the absence of Aβ pathology in cognitively normal individuals or MCI patients.^28^ Hence, in addition to the Aβ‐dependent‐tau‐cascade hypothesis, there is still a dual‐cascade hypothesis, namely, Aβ‐independent‐tau‐cascade.

Regarding the Aβ‐independent regulators of tau in AD. Among them, lipids, particularly cholesterol, are heavily involved in the pathogenesis of AD occurrence.^29^ Cholesteryl esters increase the accumulation of p‐tau by inhibiting its proteasomal degradation. Notably, this impact of cholesteryl esters on p‐tau is independent of both APP and Aβ, as reducing cholesteryl ester levels also decreases p‐tau expressions in human induced pluripotent stem cell‐derived *APP* ^null^ neurons.^30^ In addition, the endocytic system is another vital biological pathway related to sporadic AD that independently regulates both Aβ and tau. Several genes associated with an increased risk of AD encode proteins that regulate endocytosis, such as BIN1 and PICALM.^31^ Interestingly, endocytosis‐related genes PICALM and BIN1 also regulate tau accumulation in an Aβ‐independent manner.^32^, ^33^ Recently, Oskar Hansson et al reported that tau pathology was negatively associated with cortical volumes and thickness in temporal and parietal regions independently of Aβ, suggesting the potential utility of tau‐targeting treatments in individuals with low Aβ pathology.^34^

The present study indicated that PET and CSF biomarkers, established by predefined cutoffs divided into positive (+) and negative (−) subjects, were able to separate clinically progressing from clinically stable patients. Generally, tau tangle pathology was more strongly associated than Aβ plaque pathology with cognitive impairment.^35^, ^36^ However, inconsistent with these concepts, the superiority of prediction utility of Aβ‐PET over CSF tau alone in our study may be due to a number of plausible reasons. First, tau and Aβ markers change at different points in the disease, suggesting that Aβ, instead of tau, would be an earlier marker than tau. It has been speculated that Aβ levels can be abnormal slightly earlier in the disease than tau read.^37^ Moreover, there have been several publications exploring the longitudinal accumulation of tau in the AD continuum using tau‐PET,^25^, ^26^ to exclude the risk of T− at baseline change to T+ at the follow‐up visits thereby influencing the conclusions, we further selected the subjects keeping A + T− or A − T+ all the visits and still found the similar results. Second, in most cases, as aforementioned Aβ has been considered the initial insult that drives both the tau pathology and tau‐mediated neurodegeneration in the AD continuum.^38^, ^39^ That is, several previous studies reported that tau was highly related to cognitive impairment probably under the Aβ pathology drive, and may be parallel to A + T+ subjects. However, the focus point of this study is selecting the participants with tau pathology independently of Aβ accumulation (A − T+ subjects) from beginning to end, which may be the main potential cause leading to this discrepancy, indicating tau alone pathology sparse with Aβ exists different roles related to the longitudinal memory decline. Furthermore, there are natural fluctuations or variations in the production, secretion, and degradation of CSF proteins,^40^ making the CSF tau levels hard to control in a certain context. Third, we used the Aβ‐PET as a representation for A+, but CSF p‐tau indicates the T+ in this study. The difference between neuroimaging and CSF may lead to uncertainty in results. Moreover, the sample was heavily weighted toward A + T− (*n* = 134) compared to A − T+ (*n* = 49). The unbalance in sample size between these two groups will have some impact on these results. Finally, the cascade of A + T− biomarkers can follow a typical AD pathway, which is the most represented in the AD continuum. However, the relationships of patterns with Aβ/tau, such as A − T+ status, identify another large population without the classical presence of AD pathology, referred to as nontypical AD or non‐AD dementia, which has different molecular dementia mechanisms and discrepant time course and prediction patterns. Namely, the flow diagrams of pattern pathways and longitudinal cognition trajectory are probably different in the ATN framework (e.g., A + T− and A − T+) between typical AD and nontypical AD.^41^

Notably, a higher percentage of AD participants carried the APOE ε4 allele, the largest genetic risk factor for AD. Based on the cross‐sectional and longitudinal memory test studies using linear regression and linear mixed models, we found that A+ subjects with carriers of the APOE ε4 allele have abnormally low memory function in the same test domains compared to the T+ with APOE ε4 allele carriers, suggesting APOE ε4 carriers develop serious impairment of cognition even lead to AD dementia with the Aβ pathology background. Nonetheless, the impact of APOE ε4 on cognition function in Aβ‐positive subjects has been displayed to vary widely between different cohorts,^42^ indicating that the Aβ‐independent effects of APOE ε4 on tau aggregation and cognitive decline are ambiguous and cohort‐dependent. Additionally, a study according to the ADNI data instead found an interaction of APOE ε4 status and sex on entorhinal tau load, including CSF tau (p‐tau, t‐tau) and brain tau PET.^43^ However, the available data are limited and did not involve memory assessment in this cross‐sectional analysis. Similarly, APOE ε4 status is also associated with an increased rate of Aβ accumulation in participants that were Aβ‐negative at baseline, but not among the Aβ‐positive participants.^44^ These studies point out that the relationship between Aβ/tau pathologies and APOE ε4 is inextricably linked. In this longitudinal study, we showed that APOE ε4 carriers in Aβ‐positive subjects were associated with a faster cognitive decline, independent of tau load, providing evidence that APOE ε4 with Aβ pathology exhibit an increased rate of dementia over time. Moreover, Ruben et al found the effect of APOE ε4 on the tau accumulation dependent Aβ pathology exists.^45^ By comparison, one study reported that APOE ε4 was associated with high levels of tau‐PET deposition in the temporal lobe independent of Aβ status.^46^ Therefore, the real interaction between these indexes still needs more studies to confirm further.

The main advantage of this study was its reliance on a large size of longitudinal data on CSF biomarkers and numerous scans using a newly available PET tracer for Aβ pathology and cognitive evaluations. Additionally, we acknowledge the limitations of this ADNI longitudinal study, which possibly influence the interpretation of these results. First, we used the Aβ‐PET read analysis as a surrogate for Aβ pathology (A) in this study, the limitation of the visual PET method is inevitable as it is partly subjective and reader‐dependent. Second, to our knowledge, PET and CSF biomarkers of Aβ or tau pathology show good concordance most of the time across the AD spectrum, these associations may be lost when considering individual patient groups or different stages of Aβ or tau pathology.^47^ Due to the sample consideration, we only applied Aβ‐PET and CSF tau in this study to reflect the A and T components. Presently, the best evaluation manners of AD‐related pathologies within the ATN system are controversial due to a lack of extensive evidence on clinical validity and utility.^48^ A widely accepted methodology of a “surrogate biomarker” for “disease modification” in AD is yet to be established. Generally, well‐validated neuroimaging and CSF exist for ATN systems, with a trend—albeit not yet fully established—toward CSF biomarkers becoming positive earlier than PET.^49^ Several studies pointed out that the CSF Aβ_42_/Aβ_40_ and evidence of cortical Aβ deposition on Aβ‐PET—both become positive during the preclinical stage of AD.^19^ Third, the ADNI preanalytical protocol contains a series of handling steps, which may not have been precisely replicated in this study. Moreover, the sample was heavily weighted toward MCI and unbalanced within each group. The sampling imbalance and potential biases pose a significant danger to the validity of research findings by distorting results toward a particular segment of a target population, leading to skewed results and potentially flawed conclusions.

## 5 CONCLUSIONS

Our study points out that both Aβ‐PET and CSF tau are related to cognitive decline across the AD clinical spectrum. Of note, currently available Aβ‐PET deposition alone without tau pathology (A + T−) measure is an independent reliable predictor of longitudinal cognitive decline but may nonetheless forecast different statuses of dementia progression. However, tau accumulation sparing with Aβ pathology background (A − T+) was not enough to be an independent predictor of cognitive worsening, indicating tau needs accompanied by Aβ‐driven together.

## AUTHOR CONTRIBUTIONS

HJH, XYY, and JLC analyzed the data. EM, TJ, SJQ, and RXA searched the literature. YZ, JLH, and WWW made substantial contributions to conception and replenished the required data. XSW and CLX were involved in drafting the manuscript. JNH participated in the revision of the current manuscript.

## FUNDING INFORMATION

Supported by the Projects of the National Science Foundation of China (Nos. 82001363, 81600977, and 82271469) and the Projects of the Natural Science Foundation of Zhejiang Province (LQ23H090007, Y19H090059, and LZ23H090001), and the Projects of the Wenzhou City Committee of Science and Technology (No. Y20220164). Moreover, the study was also funded by the Leading Innovative and Entrepreneur Team Introduction Program of Zhejiang (2023R01002).

## CONFLICT OF INTEREST STATEMENT

The authors report no disclosures relevant to the manuscript.

## Supporting information


Data S1

## Data Availability

ADNI has made publicly available the data used in this study in the Neuroimaging Laboratory (LONI) database (adni.loni.usc.edu).

## References

[1] Knopman DS , Amieva H , Petersen RC , et al. Alzheimer disease. Nat Rev Dis Primers. 2021;7(1):33. 33986301 10.1038/s41572-021-00269-y PMC8574196

[2] Hampel H , Hardy J , Blennow K , et al. The amyloid‐beta pathway in Alzheimer's disease. Mol Psychiatry. 2021;26(10):5481‐5503. 34456336 10.1038/s41380-021-01249-0 PMC8758495

[3] Long JM , Holtzman DM . Alzheimer disease: An update on pathobiology and treatment strategies. Cell. 2019;179(2):312‐339. 31564456 10.1016/j.cell.2019.09.001 PMC6778042

[4] Panza F , Lozupone M , Logroscino G , Imbimbo BP . A critical appraisal of amyloid‐beta‐targeting therapies for Alzheimer disease. Nat Rev Neurol. 2019;15(2):73‐88. 30610216 10.1038/s41582-018-0116-6

[5] Romoli M , Sen A , Parnetti L , Calabresi P , Costa C . Amyloid‐beta: a potential link between epilepsy and cognitive decline. Nat Rev Neurol. 2021;17(8):469‐485. 34117482 10.1038/s41582-021-00505-9

[6] Sepulcre J , Grothe MJ , Sabuncu M , et al. Hierarchical organization of tau and amyloid deposits in the cerebral cortex. JAMA Neurol. 2017;74(7):813‐820. 28558094 10.1001/jamaneurol.2017.0263 PMC5710537

[7] Koss DJ , Dubini M , Buchanan H , Hull C , Platt B . Distinctive temporal profiles of detergent‐soluble and ‐insoluble tau and Abeta species in human Alzheimer's disease. Brain Res. 2018;1699:121‐134. 30102892 10.1016/j.brainres.2018.08.014

[8] Congdon EE , Sigurdsson EM . Tau‐targeting therapies for Alzheimer disease. Nat Rev Neurol. 2018;14(7):399‐415. 29895964 10.1038/s41582-018-0013-z PMC6463489

[9] Baek MS , Cho H , Lee HS , et al. Temporal trajectories of in vivo tau and amyloid‐beta accumulation in Alzheimer's disease. Eur J Nucl Med Mol Imaging. 2020;47(12):2879‐2886. 32350558 10.1007/s00259-020-04773-3

[10] Villemagne VL , Burnham S , Bourgeat P , et al. Amyloid beta deposition, neurodegeneration, and cognitive decline in sporadic Alzheimer's disease: a prospective cohort study. Lancet Neurol. 2013;12(4):357‐367. 23477989 10.1016/S1474-4422(13)70044-9

[11] Iqbal K , Liu F , Gong CX . Tau and neurodegenerative disease: the story so far. Nat Rev Neurol. 2016;12(1):15‐27. 26635213 10.1038/nrneurol.2015.225

[12] van der Kant R , Goldstein LSB , Ossenkoppele R . Amyloid‐beta‐independent regulators of tau pathology in Alzheimer disease. Nat Rev Neurosci. 2020;21(1):21‐35. 31780819 10.1038/s41583-019-0240-3

[13] Bittner T , Zetterberg H , Teunissen CE , et al. Technical performance of a novel, fully automated electrochemiluminescence immunoassay for the quantitation of β‐amyloid (1‐42) in human cerebrospinal fluid. Alzheimers Dement. 2016;12(5):517‐526. 26555316 10.1016/j.jalz.2015.09.009

[14] Karikari TK , Pascoal TA , Ashton NJ , et al. Blood phosphorylated tau 181 as a biomarker for Alzheimer's disease: a diagnostic performance and prediction modelling study using data from four prospective cohorts. Lancet Neurol. 2020;19(5):422‐433. 32333900 10.1016/S1474-4422(20)30071-5

[15] Meyer PF , Pichet Binette A , Gonneaud J , Breitner JCS , Villeneuve S . Characterization of Alzheimer disease biomarker discrepancies using cerebrospinal fluid phosphorylated tau and AV1451 positron emission tomography. JAMA Neurol. 2020;77(4):508‐516. 31961372 10.1001/jamaneurol.2019.4749 PMC6990861

[16] Jack CR Jr , Bernstein MA , Borowski BJ , et al. Update on the magnetic resonance imaging core of the Alzheimer's disease neuroimaging initiative. Alzheimers Dement. 2010;6(3):212‐220. 20451869 10.1016/j.jalz.2010.03.004 PMC2886577

[17] Jagust WJ , Bandy D , Chen K , et al. The Alzheimer's disease neuroimaging initiative positron emission tomography core. Alzheimers Dement. 2010;6(3):221‐229. 20451870 10.1016/j.jalz.2010.03.003 PMC2920531

[18] Hampel H , Cummings J , Blennow K , Gao P , Jack CR Jr , Vergallo A . Developing the ATX(N) classification for use across the Alzheimer disease continuum. Nat Rev Neurol. 2021;17(9):580‐589. 34239130 10.1038/s41582-021-00520-w

[19] Hansson O , Seibyl J , Stomrud E , et al. CSF biomarkers of Alzheimer's disease concord with amyloid‐beta PET and predict clinical progression: a study of fully automated immunoassays in BioFINDER and ADNI cohorts. Alzheimers Dement. 2018;14(11):1470‐1481. 29499171 10.1016/j.jalz.2018.01.010 PMC6119541

[20] Franzmeier N , Rubinski A , Neitzel J , Ewers M , Alzheimer's Disease Neuroimaging I . The BIN1 rs744373 SNP is associated with increased tau‐PET levels and impaired memory. Nat Commun. 2019;10(1):1766. 30992433 10.1038/s41467-019-09564-5 PMC6467911

[21] Griciuc A , Serrano‐Pozo A , Parrado AR , et al. Alzheimer's disease risk gene CD33 inhibits microglial uptake of amyloid beta. Neuron. 2013;78(4):631‐643. 23623698 10.1016/j.neuron.2013.04.014 PMC3706457

[22] Bucci M , Chiotis K , Nordberg A , Alzheimer's Disease Neuroimaging Initiative . Alzheimer's disease profiled by fluid and imaging markers: tau PET best predicts cognitive decline. Mol Psychiatry. 2021;26(10):5888‐5898. 34593971 10.1038/s41380-021-01263-2 PMC8758489

[23] Reimand J , Collij L , Scheltens P , Bouwman F , Ossenkoppele R , Alzheimer's Disease Neuroimaging Initiative . Association of amyloid‐beta CSF/PET discordance and tau load 5 years later. Neurology. 2020;95(19):e2648‐e2657. 32913020 10.1212/WNL.0000000000010739 PMC7963352

[24] Mattsson‐Carlgren N , Andersson E , Janelidze S , et al. Abeta deposition is associated with increases in soluble and phosphorylated tau that precede a positive tau PET in Alzheimer's disease. Sci Adv. 2020;6(16):eaaz2387. 32426454 10.1126/sciadv.aaz2387 PMC7159908

[25] Jack CR Jr , Wiste HJ , Schwarz CG , et al. Longitudinal tau PET in ageing and Alzheimer's disease. Brain. 2018;141(5):1517‐1528. 29538647 10.1093/brain/awy059 PMC5917767

[26] Pontecorvo MJ , Devous MD , Kennedy I , et al. A multicentre longitudinal study of flortaucipir (18F) in normal ageing, mild cognitive impairment and Alzheimer's disease dementia. Brain. 2019;142(6):1723‐1735. 31009046 10.1093/brain/awz090 PMC6536847

[27] Ossenkoppele R , Smith R , Ohlsson T , et al. Associations between tau, Abeta, and cortical thickness with cognition in Alzheimer disease. Neurology. 2019;92(6):e601‐e612. 30626656 10.1212/WNL.0000000000006875 PMC6382060

[28] Crary JF , Trojanowski JQ , Schneider JA , et al. Primary age‐related tauopathy (PART): a common pathology associated with human aging. Acta Neuropathol. 2014;128(6):755‐766. 25348064 10.1007/s00401-014-1349-0 PMC4257842

[29] Di Paolo G , Kim TW . Linking lipids to Alzheimer's disease: cholesterol and beyond. Nat Rev Neurosci. 2011;12(5):284‐296. 21448224 10.1038/nrn3012 PMC3321383

[30] van der Kant R , Langness VF , Herrera CM , et al. Cholesterol metabolism is a druggable Axis that independently regulates tau and amyloid‐beta in iPSC‐derived Alzheimer's disease neurons. Cell Stem Cell. 2019;24(3):363‐375 e369. 30686764 10.1016/j.stem.2018.12.013 PMC6414424

[31] Maninger JK , Nowak K , Goberdhan S , O'Donoghue R , Connor‐Robson N . Cell type‐specific functions of Alzheimer's disease endocytic risk genes. Philos Trans R Soc Lond Ser B Biol Sci. 1899;2024(379):20220378. 10.1098/rstb.2022.0378 PMC10874703 38368934

[32] Sartori M , Mendes T , Desai S , et al. BIN1 recovers tauopathy‐induced long‐term memory deficits in mice and interacts with tau through Thr(348) phosphorylation. Acta Neuropathol. 2019;138(4):631‐652. 31065832 10.1007/s00401-019-02017-9 PMC6778065

[33] Moreau K , Fleming A , Imarisio S , et al. PICALM modulates autophagy activity and tau accumulation. Nat Commun. 2014;5:4998. 25241929 10.1038/ncomms5998 PMC4199285

[34] Wuestefeld A , Pichet Binette A , Berron D , et al. Age‐related and amyloid‐beta‐independent tau deposition and its downstream effects. Brain. 2023;146(8):3192‐3205. 37082959 10.1093/brain/awad135 PMC10393402

[35] Mielke MM , Hagen CE , Xu J , et al. Plasma phospho‐tau181 increases with Alzheimer's disease clinical severity and is associated with tau‐ and amyloid‐positron emission tomography. Alzheimers Dement. 2018;14(8):989‐997. 29626426 10.1016/j.jalz.2018.02.013 PMC6097897

[36] Saha P , Sen N . Tauopathy: a common mechanism for neurodegeneration and brain aging. Mech Ageing Dev. 2019;178:72‐79. 30668956 10.1016/j.mad.2019.01.007 PMC6377302

[37] Jack CR Jr , Knopman DS , Jagust WJ , et al. Hypothetical model of dynamic biomarkers of the Alzheimer's pathological cascade. Lancet Neurol. 2010;9(1):119‐128. 20083042 10.1016/S1474-4422(09)70299-6 PMC2819840

[38] Bloom GS . Amyloid‐beta and tau: the trigger and bullet in Alzheimer disease pathogenesis. JAMA Neurol. 2014;71(4):505‐508. 24493463 10.1001/jamaneurol.2013.5847 PMC12908160

[39] Kent SA , Spires‐Jones TL , Durrant CS . The physiological roles of tau and Abeta: implications for Alzheimer's disease pathology and therapeutics. Acta Neuropathol. 2020;140(4):417‐447. 32728795 10.1007/s00401-020-02196-w PMC7498448

[40] Lucey BP , Fagan AM , Holtzman DM , Morris JC , Bateman RJ . Diurnal oscillation of CSF Abeta and other AD biomarkers. Mol Neurodegener. 2017;12(1):36. 28478762 10.1186/s13024-017-0161-4 PMC5421331

[41] Yang Z , Nasrallah IM , Shou H , et al. A deep learning framework identifies dimensional representations of Alzheimer's disease from brain structure. Nat Commun. 2021;12(1):7065. 34862382 10.1038/s41467-021-26703-z PMC8642554

[42] Insel PS , Weiner M , Mackin RS , et al. Determining clinically meaningful decline in preclinical Alzheimer disease. Neurology. 2019;93(4):e322‐e333. 31289148 10.1212/WNL.0000000000007831 PMC6669933

[43] Liu M , Paranjpe MD , Zhou X , et al. Sex modulates the ApoE epsilon4 effect on brain tau deposition measured by (18)F‐AV‐1451 PET in individuals with mild cognitive impairment. Theranostics. 2019;9(17):4959‐4970. 31410194 10.7150/thno.35366 PMC6691387

[44] Ge T , Sabuncu MR , Smoller JW , Sperling RA , Mormino EC , For the Alzheimer's Disease Neuroimaging Initiative . Dissociable influences of APOE epsilon4 and polygenic risk of AD dementia on amyloid and cognition. Neurology. 2018;90(18):e1605‐e1612. 29592889 10.1212/WNL.0000000000005415 PMC5931806

[45] Smith R , Strandberg O , Mattsson‐Carlgren N , et al. The accumulation rate of tau aggregates is higher in females and younger amyloid‐positive subjects. Brain. 2020;143(12):3805‐3815. 33439987 10.1093/brain/awaa327 PMC7805812

[46] Mattsson N , Ossenkoppele R , Smith R , et al. Greater tau load and reduced cortical thickness in APOE epsilon4‐negative Alzheimer's disease: a cohort study. Alzheimers Res Ther. 2018;10(1):77. 30086796 10.1186/s13195-018-0403-x PMC6081879

[47] Illan‐Gala I , Pegueroles J , Montal V , et al. Challenges associated with biomarker‐based classification systems for Alzheimer's disease. Alzheimers Dement. 2018;10:346‐357. 10.1016/j.dadm.2018.03.004 PMC6114028 30175226

[48] Dubois B , Villain N , Frisoni GB , et al. Clinical diagnosis of Alzheimer's disease: recommendations of the international working group. Lancet Neurol. 2021;20(6):484‐496. 33933186 10.1016/S1474-4422(21)00066-1 PMC8339877

[49] Xiong X , He H , Ye Q , et al. Alzheimer's disease diagnostic accuracy by fluid and neuroimaging ATN framework. CNS Neurosci Ther. 2024;30(2):e14357. 37438991 10.1111/cns.14357 PMC10848089

