# Supplementary material for: Amyloid‐β but not tau accumulation is strongly associated with longitudinal cognitive decline
Source: CNS Neurosci Ther. 2024 Jul 16;30(7):e14860. doi: 10.1111/cns.14860 (PMC11251873; doi:10.1111/cns.14860)
Supplement: Supplementary file 1 — Data S1 [file CNS-30-e14860-s001.zip › cns14860-sup-0001-SupinfoS1/cns14860-sup-0001-SupinfoS1.pdf]

# Supplementary Materials

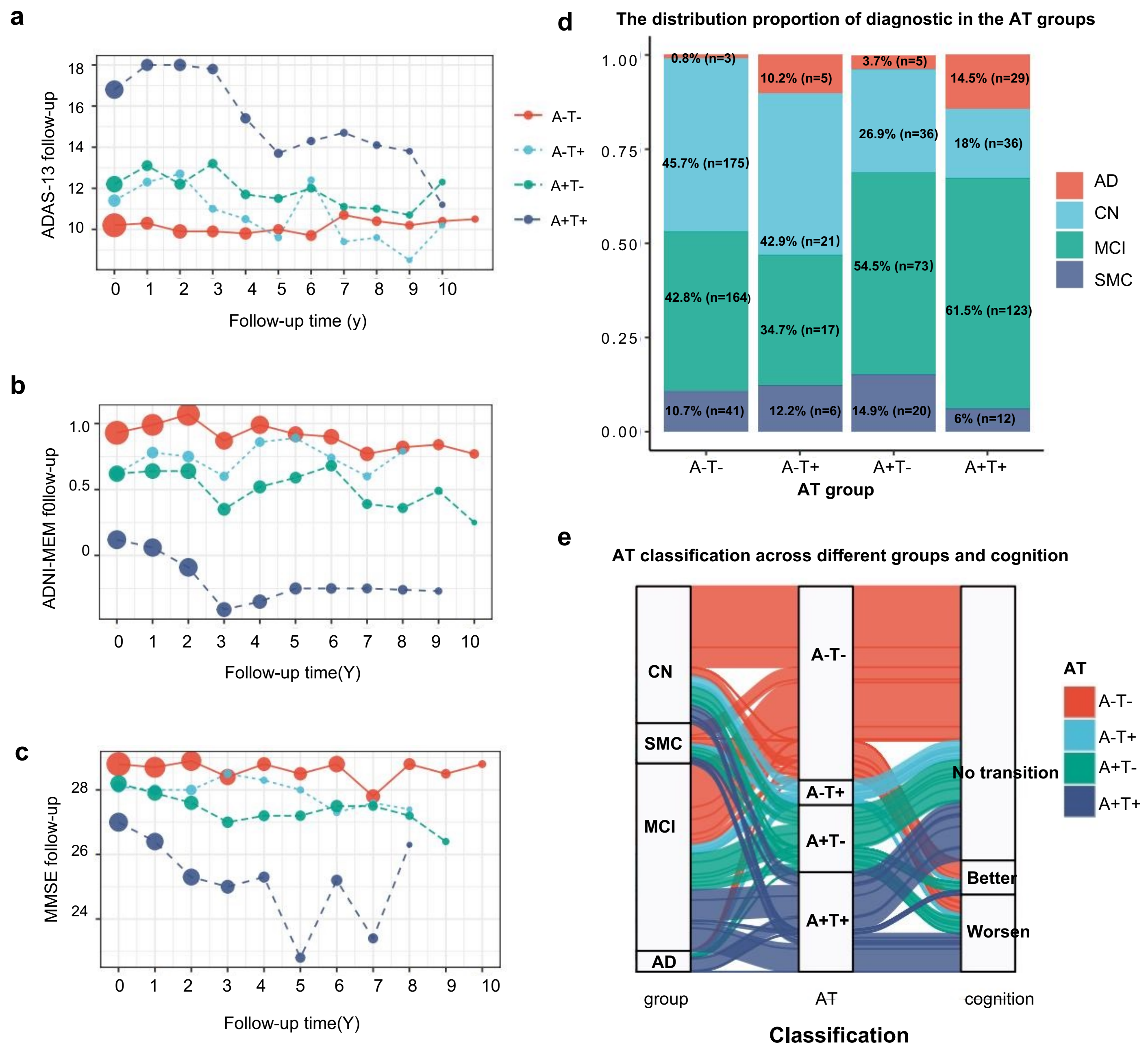
a
ADAS-13 follow-up
Follow-up time (y)
A-T-
A-T+
A+T-
A+T+
b
ADNI-MEM follow-up
Follow-up time(Y)
c
MMSE follow-up
Follow-up time(Y)
d
The distribution proportion of diagnostic in the AT groups
0.8% (n=3)
45.7% (n=175)
42.8% (n=164)
10.7% (n=41)
10.2% (n=5)
42.9% (n=21)
34.7% (n=17)
12.2% (n=6)
3.7% (n=5)
26.9% (n=36)
54.5% (n=73)
14.9% (n=20)
14.5% (n=29)
18% (n=36)
61.5% (n=123)
6% (n=12)
AD
CN
MCI
SMC
AT group
e
AT classification across different groups and cognition
CN
SMC
MCI
AD
A-T-
A-T+
A+T-
A+T+
No transition
Better
Worsen
group
AT
cognition
Classification

**Supple Fig. 1**

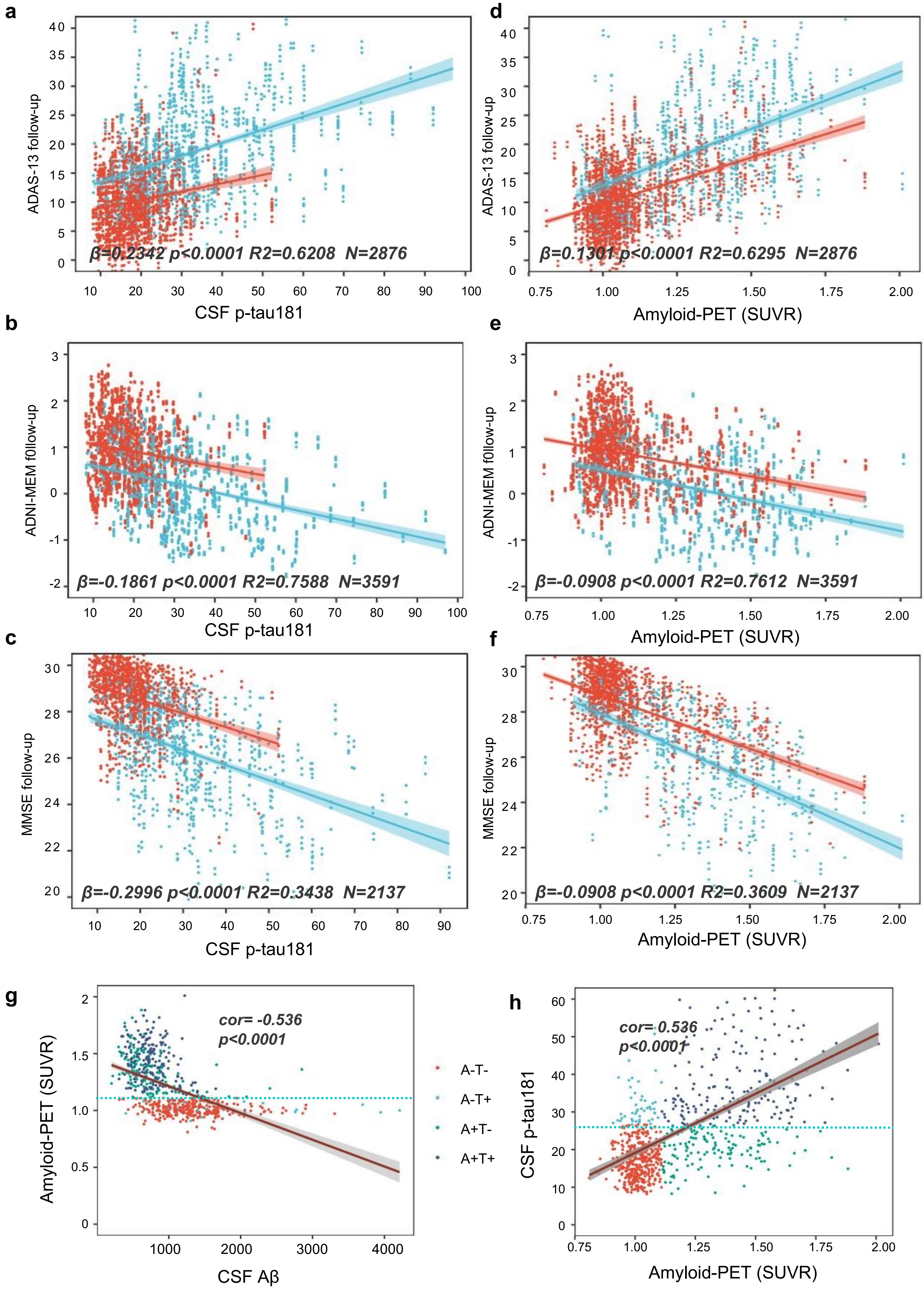
a
ADAS-13 follow-up
β=0.2342 p<0.0001 R2=0.6208 N=2876
CSF p-tau181
d
ADAS-13 follow-up
β=0.1301 p<0.0001 R2=0.6295 N=2876
Amyloid-PET (SUVR)
b
ADNI-MEM f0llow-up
β=-0.1861 p<0.0001 R2=0.7588 N=3591
CSF p-tau181
e
ADNI-MEM f0llow-up
β=-0.0908 p<0.0001 R2=0.7612 N=3591
Amyloid-PET (SUVR)
c
MMSE follow-up
β=-0.2996 p<0.0001 R2=0.3438 N=2137
CSF p-tau181
f
MMSE follow-up
β=-0.0908 p<0.0001 R2=0.3609 N=2137
Amyloid-PET (SUVR)
g
Amyloid-PET (SUVR)
cor= -0.536
p<0.0001
CSF Aβ
A-T-
A-T+
A+T-
A+T+
h
CSF p-tau181
cor= 0.536
p<0.0001
Amyloid-PET (SUVR)

**Supple Fig. 2**

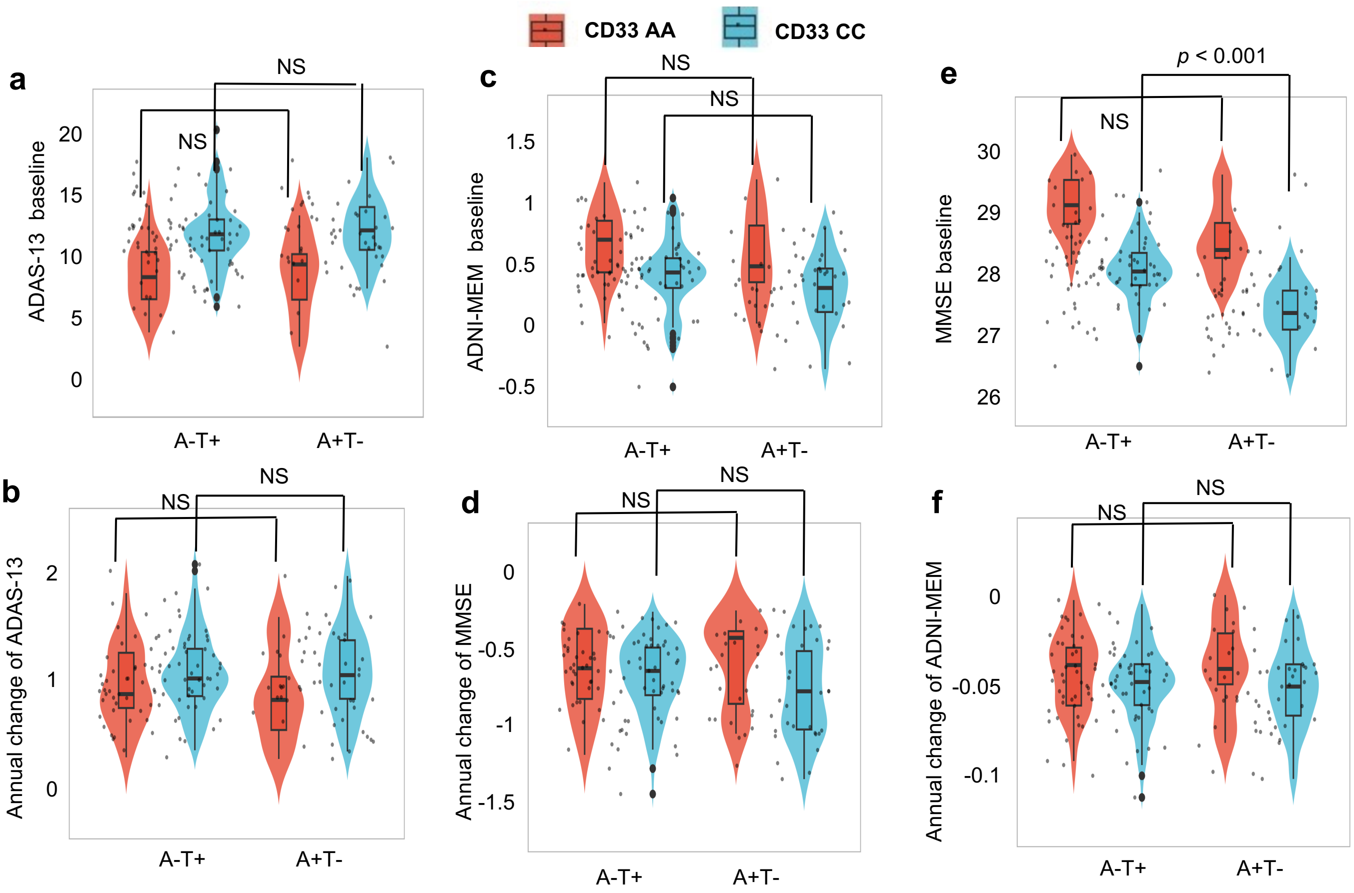

CD33 AA
CD33 CC
a
ADAS-13 baseline
NS
NS
A-T+
A+T-
b
Annual change of ADAS-13
NS
NS
c
ADNI-MEM baseline
NS
NS
d
Annual change of MMSE
NS
NS
e
MMSE baseline
$p$ < 0.001
NS
f
Annual change of ADNI-MEM
NS
NS


**Supple Fig. 3**

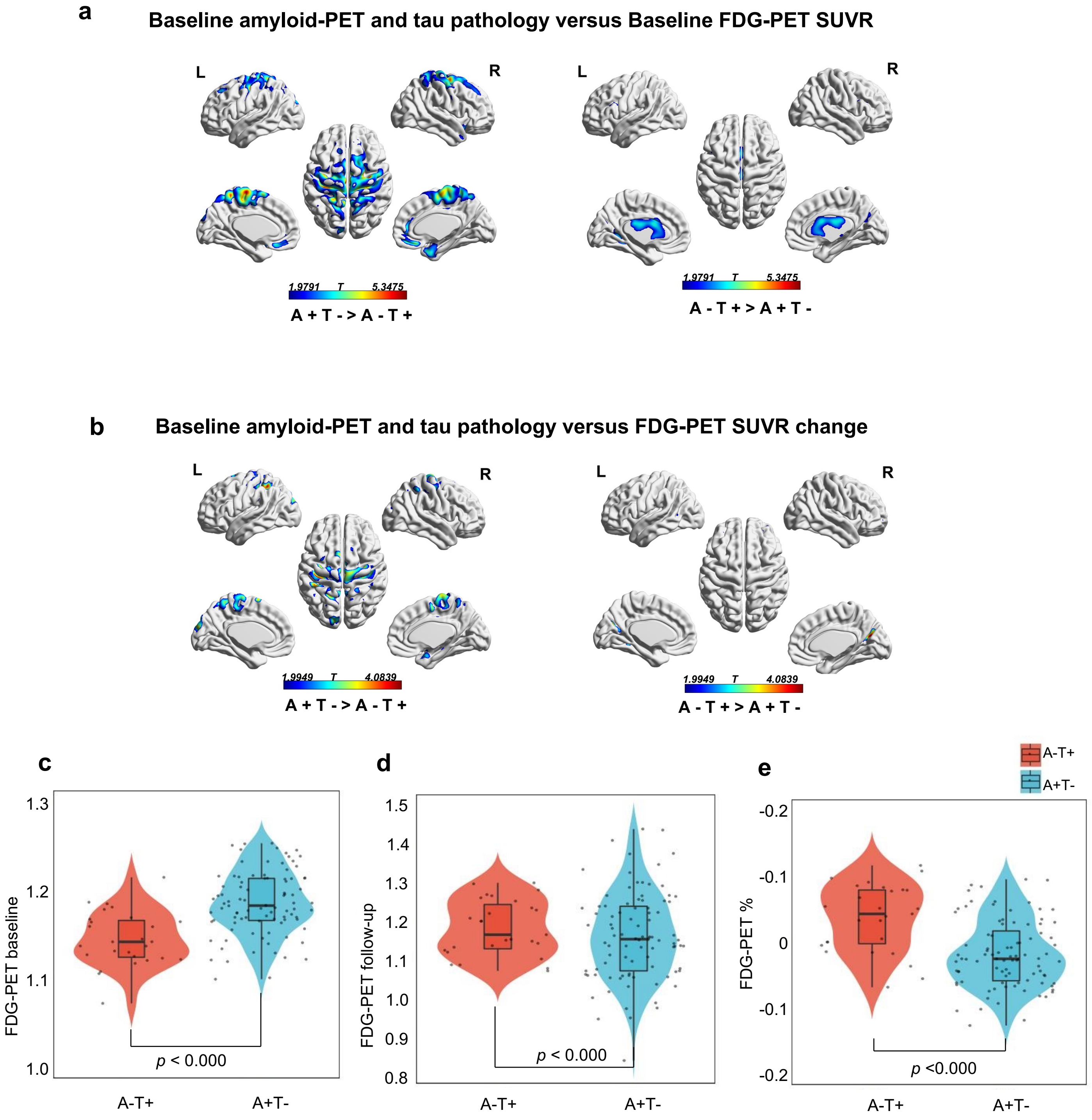
a
Baseline amyloid-PET and tau pathology versus Baseline FDG-PET SUVR
L
R
1.9791 T 5.3475
A + T - > A - T +
A - T + > A + T -
b
Baseline amyloid-PET and tau pathology versus FDG-PET SUVR change
1.9949 T 4.0839
A + T - > A - T +
A - T + > A + T -
c
d
e
A-T+
A+T-
FDG-PET baseline
FDG-PET follow-up
FDG-PET %
p < 0.000
p < 0.000
p <0.000

Supple Fig. 4

**Supple Table 1: Baseline participant characteristics of the ADNI participants (N=1272) according to clinical diagnosis**

| Cohort, n (%) | CN | SMC | MCI | AD | *p* value |
|---|---|---|---|---|---|
| **Group SUM** | 364 (31.1%) | 96 (8.2%) | 544 (49.4%) | 168 (14.3%) | |
| **ADNI 1** | 0 | 0 (0%) | 7 (1.8%) | 1 (2.3%) | |
| **ADNI GO** | 0 | 0 (0%) | 92 (24%) | 0 (0%) | |
| **ADNI-2** | 162 (44.5%) | 99 (100%) | 314 (57.7%) | 131 (78.0%) | |
| **ADNI-3** | 202 (55.5%) | 0 (0%) | 106 (19.5%) | 36 (21.4%) | |
| **Age at baseline, (years)** | 71.2 (67.1, 76.2)$^{d}$ | 71.9 (67.4, 76.8) | 71.5 (66.3, 76.8)$^{d}$ | 74.5 (68.6, 79.7)$^{a,c}$ | 0.002 |
| **Weight (kg)** | 73.7 (63, 86.8)$^{c}$ | 79.6 (65.2, 88.1)$^{d}$ | 77.1 (68.1, 87.1)$^{a,d}$ | 72.2 (62.3, 83.8)$^{b,c}$ | 0.000 |
| **Sex, n (%)** | | | | | |
| **male** | 148 (40.7%) | 39 (40.6%) | 300 (55.1%) | 103 (61.3%) | |
| **female** | 216 (59.3%) | 57 (59.4%) | 224 (44.9%) | 65 (38.7%) | |
| **Education (years)** | 17 (16,18) | 17 (16,18) | 16 (14,18) | 16 (14,18) | 0.000 |
| **APOE $\varepsilon4^{+/-}$** | 113 (30%) (n=360) | 32 (33.3%) | 254 (46.7%) (n=528) | 112 (66.7%) (n=166)* | 0.000 |
| **Cognition function** | | | | | |
| **MMSE** | 29 (29, 30)$^{c,d}$ | 29 (28, 30)$^{c,d}$ | 28 (27, 29)$^{a,b,d}$ | 23 (21, 25)$^{a,b,c}$ | 0.000 |
| **ADAS_13** | 8 (5, 11)$^{c,d}$ | 8 (5, 12)$^{c,d}$ | 14 (10, 19)$^{a,b,d}$ | 30 (24, 36)$^{a,b,d}$ | 0.000 |
| **ADNI_MEM** | 1.03 (0.61, 1.43)$^{c,d}$ | 1.07 (0.73, 1.55)$^{c,d}$ | 0.35 (-0.09, 0.79)$^{a,b,d}$ | -0.90 (-1.19, -0.53)$^{a,b,d}$ | 0.000 |
| **Fluid biomarkers** | | | | | |
| **CSF Aβ42 pg /ml** | 1150 (828, 1571)$^{c,d}$ (n=325) | 1086 (780.3, 1786)$^{c,d}$ (n=71) | 819.6 (622.7, 1175)$^{a,b,d}$ (n=473) | 593.8 (455.6, 756.0)$^{a,b,c}$ (n=162) | 0.000 |
| **CSF Aβ40 pg /ml** | 18490 (14815, 21995) (n=233) | NA | 17290 (14985, 21795) (n=100) | 16165 (12653,18713) (n=34) | 0.035 |
| **CSF Aβ42/40** | 0.076 (0.050, 0.093)$^{c,d}$ (n=233) | NA | 0.054 (0.034, 0.087)$^{a,d}$ (n=100) | 0.034 (0.031, 0.050)$^{a,c}$ (n=34) | 0.000 |
| **CSF p-Tau pg/ml** | 19.0 (14.7, 24.7)$^{c,d}$ | 19.7 (14.7, 26.5)$^{b,d}$ | 22.8 (16.9, 32.0)$^{a,b,d}$ | 32.7 (25.3, 45.5)$^{a,b,c}$ | 0.000 |
| **CSF p-Tau >26.64 pg/ml (%)** | 76 (20.9%) | 23 (24%) | 207 (38.1%) | 117 (69.6%) | |
| **CSF T-Tau pg /ml** | 210.2 (169.9, 270.7)$^{c,d}$ | 219.5 (168.8, 301.9)$^{d}$ | 243.8 (186.7, 325.7)$^{a,d}$ | 329(266.8, 440)$^{a,b,c}$ (n=167) | 0.000 |
| **Plasma P-tau181 pg /ml** | 13.4 (8.9, 19.0)$^{c,d}$ (n=162) | 13.3 (9.3, 18.0)$^{c,d}$ | 15.5 (10.6, 22.9)$^{a,b,d}$ (n=435) | 23.0 (17.8, 28.5)$^{a,b,c}$ (n=129) | 0.000 |
| **PET_SUVR, mean (SD)** | | | | | |
| **Amyloid_PET** | 1.04 (0.99,1,17)$^{c,d}$ (n=363)* | 1.07 (1.01, 1.23)$^{d}$ | 1.16 (1.01, 1.40)$^{a,d}$ (n=537) | 1.45 (1.28, 1.58)$^{a,b,c}$ (n=165) | 0.000 |
| **Amyloid_SUVR >1.1** | 110 (30.2%) | 35 (36.5%) | 292 (53.7%) | 144 (85.7%) | 0.000 |

ADNI, Alzheimer's Disease Neuroimaging Initiative; CN: cognitively normal; SMC: subjective memory complaints; MCI: mild cognitive impairment; AD: Alzheimer' disease; APOE, apolipoprotein E; Aβ42: β-amyloid; APOE-/+ non-carriers and carriers of the APOE ε4 allele; MMSE: Mini-Mental State Examination; ADAS_13: 13-item Alzheimer's Disease Assessment Scale-cognitive subscale (ADAS-cog13); ADNI_MEM: Alzheimer's Disease Neuroimaging Initiative Memory Score; tTau: total tau; pTau: phosphorylated tau; CSF, cerebrospinal fluid; PET: positron emission tomography; SUVR: standardized uptake value ratio (using a composite reference region) corrected for partial volume errors. Data are shown as P50 (P25, P75) or n (%). Continuous variables were compared with the Kruskal-wallis test by with Mann–Whitney U statistic Tukey's post-hoc test. Categorical variables were compared with Chi-square test. Elevated brain amyloid was defined as baseline amyloid PET SUVR > 1.1; Tau status defined by CSF p-Tau > 26.64 pg/ml; *The Effective number of participants.

**Supple Table 2: Fluid and imaging biomarkers at the baseline and follow-up end points.**

| Cohort, n (%) | CN (n=270) | SMC (n=79) | MCI (n=384) | AD (n=44) | *p* value |
|---|---|---|---|---|---|
| **Follow-up time (Y)** | 4 (2.1, 6.1) | 4.8 (2.1, 8) | 4 (2, 6.0) | 2 (2, 2.1) | |
| **Fluid biomarkers baseline** | | | | | |
| **CSF p-Tau** | 19.2 (14.7,24.7) [c, d] | 20.7 (14.4,26.3) [d] | 21.8 (16.4,32.0) [a, d] | 33.6 (27.6,46.7) [a, b, c](n=43) | 0.000 |
| **Tau (%)** | 57 (21.1%) | 18 (22.8%) | 142 (37%) | 35 (79.5%) | 0.000 |
| **CSF t-Tau** | 211.9 (171.1, 276.3) [c, d] | 220.1 (163.9, 300.1) [d] | 239.9 (185.3, 325.0) [a, d] | 335.9 (298.8, 440) [a, b, c] (n=43) | 0.000 |
| **CSF Aβ42** | 1163 (847,1589) [c, d](n=237) | 1058 (756,1401) [c, d] (n=60) | 832 (641,1167) [a, b, d] (n=325) | 616 (454,768) [a, b, c] (n=42) | 0.000 |
| **Plasma P-tau181** | 13.4 (9.3, 19.0) [d] (n=139) | 14.3 (10.5, 18.4) [d] | 14.6 (10.6, 21.8) [d] (n=331) | 25.0 (17.1, 29.0) [a, b, c] (n=27) | 0.000 |
| **Fluid biomarkers follow-up end** | | | | | |
| **CSF p-Tau** | 19.7(14.9, 26.3) [c, d] | 20.8 (14.6, 27.1) [d] | 23.0(16.7, 31.9) [a, d] (n=383) | 34.1(27.4, 45.6) [a, b, c] (n=43) | 0.000 |
| **Tau (%)** | 64 (23.7%) | 20 (25.3%) | 150 (39.1%) | 35 (79.5%) | 0.000 |
| **CSF t-Tau** | 220.5 (172, 289) [c, d] (n=269) | 232.8 (170.7, 302.5) [d] | 250.8 (187, 331.3) [a, d] (n=383) | 335.9 (298, 438) [a, b, c] (n=43) | 0.000 |
| **CSF Aβ42** | 1105 (788, 1547) [c, d] (n=236) | 1106 (764,1451) [c, d] (n=68) | 806 (614,1183) [a, b, d] (n=340) | 601(450,762) [a, b, c] | 0.000 |
| **Plasma P-tau181** | 13.9 (10, 21) [d] (n=139) | 14.7(10.4, 20.2) [d] | 16.2 (11.1, 23.3) [d] (n=331) | 25.5(19.3, 30.0) [a, b, c] (n=27) | 0.000 |
| **PET_SUVR baseline, mean (SD)** | | | | | |
| **Amyloid_PET** | 1.03 (0.99,1,12) [b, c, d] | 1.08 (1.01,1.24) [a, d] | 1.14 (1.01,1.38) [a, d] (n=377) | 1.47 (1.28,1.64) [a, b, c] (n=43) | 0.000 |
| **Aβ+ (%)** | 72 (26.7%) | 32 (40.5%) | 196 (51%) (n=377) | 35 (79.5%) (n=43) | 0.000 |
| **PET_SUVR follow-up end, mean (SD)** | | | | | |
| **Amyloid PET SUVR** | 1.05 (0.99, 1,23) [c, d] | 1.11(1.02, 1.33) [d] | 1.21 (1.02, 1.46) [a, d] (n=380) | 1.46 (1.28, 1.61) [a, b, c] (n=43) | 0.000 |
| **Aβ+ (%)** | 95 (35.2%) | 38 (48.1%) | 221 (57.6%) | 35 (79.5%) | 0.000 |
| **Convert to dementia** | 45 (16.7%) | 27 (34.2%) | 80 (20.8%) | 0 (0) | 0.000 |

ADNI: Alzheimer's Disease Neuroimaging Initiative; CN: cognitively normal; SMC: subjective memory complaints; MCI: mild cognitive impairment; AD: Alzheimer' disease; tTau: total tau; p Tau: phosphorylated tau; CSF, cerebrospinal fluid; PET: positron emission tomography; SUVR: standardized uptake value ratio. Data are shown as P50 (P25, P75) or n (%). Continuous data were evaluated using Kruskal-Wallis, categorical data were evaluated by Chi-square test (χ2), and post hoc comparisons were adjusted by Bonferroni. Aβ positivity (A+) was defined as FBP and FBB SUVR exceeding 1.11 and 1.08, respectively.; Tau positivity (T+) was defined as CSF p-Tau>26.64 pg/ml; *p* values are given overall among CN, SMC, MCI, AD. a (CN) b (SMC) c (MCI) d (AD) *p-value* < 0.05

**Supple Table 3: Temporal trajectories of memory tests in each AT group**

| Memory tests | Follow-up visits (Years) | | | | | | | | | | | |
|---|---|---|---|---|---|---|---|---|---|---|---|---|
| **ADAS_13** | **0** | **1** | **2** | **3** | **4** | **5** | **6** | **7** | **8** | **9** | **10** | **11** |
| **A-T-** | 9.6(383) | 10.3 (228) | 9.9 (336) | 9.9 (173) | 9.8 (199) | 10 (105) | 9.7 (123) | 10.7 (75) | 10.4 (71) | 10.2 (43) | 10.4 (36) | 10.5 (15) |
| **A-T+** | 12.2 (49) | 12.3 (43) | 12.7 (45) | 11 (21) | 10.5 (26) | 9.6 (16) | 12.4 (13) | 9.4 (9) | 9.6 (9) | 8.5 (4) | 10.2 (5) | |
| **A+T-** | 12.5 (134) | 13.1 (124) | 12.2 (125) | 13.2 (61) | 11.7 (65) | 11.5 (45) | 12 (39) | 11.1 (22) | 11 (28) | 10.7 (12) | 12.3 (8) | |
| **A+T+** | 17.9 (200) | 18 (185) | 18 (191) | 17.8 (103) | 15.4 (94) | 13.7 (50) | 14.3 (31) | 14.7 (22) | 14.1 (21) | 13.8 (8) | 11.2 (5) | |
| **ADNI_MEM** | **0** | **1** | **2** | **3** | **4** | **5** | **6** | **7** | **8** | **9** | **10** | |
| **A-T-** | 0.93 (375) | 0.99 (295) | 1.07 (337) | 0.87 (170) | 0.99(181) | 0.92 (116) | 0.9 (119) | 0.77 (90) | 0.82 (74) | 0.84 (39) | 0.77(22) | |
| **A-T+** | 0.62(48) | 0.78 (43) | 0.75 (44) | 0.6 (19) | 0.86 (23) | 0.89 (18) | 0.74 (14) | 0.6 (12) | 0.79 (8) | 1.56 (3) | | |
| **A+T-** | 0.62 (132) | 0.64 (115) | 0.64 (121) | 0.35 (62) | 0.52 (65) | 0.59 (46) | 0.68 (40) | 0.39 (25) | 0.36 (27) | 0.49 (12) | 0.25 (6) | |
| **A+T+** | 0.12 (198) | 0.06 (181) | -0.09 (190) | -0.41(100) | -0.35(89) | -0.25 (52) | -0.25 (32) | -0.25(25) | -0.26 (23) | -0.27 (9) | -0.09 (4) | |
| **MMSE** | **0** | **1** | **2** | **3** | **4** | **5** | **6** | **7** | **8** | **9** | **10** | **11** |
| **A-T-** | 28.8 (383) | 28.7 (261) | 28.9 (236) | 28.4 (129) | 28.8 (80) | 28.5 (71) | 28.8 (135) | 27.8 (85) | 28.8 (50) | 28.5 (23) | 28.8(12) | |
| **A-T+** | 28.1 (49) | 28 (38) | 28 (32) | 28.5 (15) | 28.3 (9) | 28 (9) | 27.3 (9) | 27.6 (9) | 27.4 (7) | | | |
| **A+T-** | 28.2 (134) | 27.9(106) | 27.6 (87) | 28.4 (42) | 27 (37) | 27.2 (31) | 27.2 (54) | 27.5 (23) | 27.2 (14) | 26.4 (9) | | |
| **A+T+** | 27 (200) | 26.4 (159) | 25.3 (145) | 25 (78) | 28.8 (80) | 25.3 (78) | 22.8 (27) | 25.2 (39) | 23.4 (27) | 26.3 (7) | | |

ADAS-Cog: Alzheimer Disease Assessment Scale-Cognitive; MMSE: Mini−Mental State Examination(range0−30); ADNI_MEM: Alzheimer Disease Neuroimaging Initiative Memory Score; Aβ positivity (A+) was defined as FBP and FBB SUVR exceeding 1.11 and 1.08, respectively.; Tau positivity (T+) was defined as CSF p-Tau>26.64 pg/ml; Data are not included if no follow-up was performed in a given year, follow-up visits of less than 5 were not recorded to reduce bias.

**Supple Table 4: The predicted values of the model were compared with different AT groups using linear mixed model and linear model**

| Group (AT) | A-T- | A-T+ | A+T- | A+T+ | Baseline cognition | APOE | R2 [i] |
|---|---|---|---|---|---|---|---|
| **Prediction of follow-up cognition β（p-value）[e]** | | | | | | | |
| **ADAS-13 follow-up** | | | | | | | |
| **β** | 0.650 (0.67) | 0.2325 (0.628) | 1.766 (<0.000) | 4.318 (<0.000) | 1.042 (<0.000) | 1.024 (<0.000) | 0.6228 |
| **Standardized β** | -0.088(0.06) | 0.023(0.628) | 0.173(<0.000) | 0.422 (<0.000) | 0.702 (<0.000) | 0.1 (<0.000) | |
| **estimated value** | 9.41 (1460) [d, c] | 9.24 (192) [d, c] | 13.33 (519) [a, b, d] | 21.01 (705) [a, b, c] | | | |
| **ADNI-MEM follow-up** | | | | | | | |
| **β** | 0.133 (0.2) | 0.024 (0.45) | -0.129 (<0.000) | -0.305 (<0.000) | 1.028 (<0.000) | -0.045 (0.01) | 0.7599 |
| **Standardized β** | 0.068 (0.07) | 0.025 (0.45) | -0.135 (<0.000) | -0.318 (<0.000) | 0.796 (<0.000) | -0.047 (0.01) | |
| **estimated value** | 0.89 (379) [b, d, c] | 0.62 (49)[d, a] | 0.59 (132) [a, d] | 0.10 (196) [a, b, c] | | | |
| **MMSE follow-up** | | | | | | | |
| **β** | 3.945 (0.005) | -0.2749 (0.28) | -0.780 (<0.000) | -2.064 (<0.000) | 0.82 (<0.000) | -0.099 (0.5) | 0.3463 |
| **Standardized β [g]** | 0.175 (0.005) | -0.081 (0.28) | -0.23 (<0.000) | -0.61 (<0.000) | 0.448 (<0.000) | -0.029 (0.5) | |
| **estimated value [h]** | 28.89 (1082) [b, d, c] | 28.61 (132) [d, c, a] | 27.99 (405) [a, b, d] | 25.55 (518) [a, b, c] | | | |
| **Prediction of baseline cognition β（p-value）[f]** | | | | | | | |
| **ADAS-13 baseline** | | | | | | | |
| **β** | 5.477 (<0.000) | 1.287 (0.005) | 1.534 (<0.000) | 5.646 (<0.000) | | 0.834 (0.001) | 0.2396 |
| **Standardized β** | -0.531 (<0.000) | 0.187 (0.005) | 0.223 (<0.000) | 0.819 (<0.000) | | 0.121 (0.001) | 0.2396 |

| | | | | | | | |
|---|---|---|---|---|---|---|---|
| **estimated value** | 9.79 (379) [b, d, c] | 11.97 (49)[d, a] | 12.70 (132) [a, d] | 18.02 (196) [a, b, c] | | | |
| **ADNI-MEM baseline** | | | | | | | |
| **β** | 1.391 (<0.000) | -0.206 (<0.000) | -0.301 (<0.000) | -0.881 (<0.000) | | -0.155 (<0.000) | 0.3029 |
| **Standardized β** | 0.611 (<0.000) | -0.215 (<0.000) | -0.314 (<0.000) | -0.919 (<0.000) | | -0.162 (<0.000) | 0.3029 |
| **estimated value** | 0.89 (379) [b, d, c] | 0.62 (49)[d, a] | 0.59 (132) [a, d] | 0.10 (196) [a, b, c] | | | |
| **MMSE baseline** | | | | | | | |
| **β** | 28.50 (<0.000) | -0.460 (0.003) | -0.245 (0.017) | -1.171 (<0.000) | | -0.230 (0.006) | 0.1745 |
| **Standardized β** | 0.369 (<0.000) | -0.249 (0.003) | -0.133 (0.017) | -0.635 (<0.000) | | -0.124 (0.006) | 0.2396 |
| **estimated value** | 28.84 (379) [b, d, c] | 28.21 (49)[d, a] | 28.18 (132) [a, d] | 27.04 (196) [a, b, c] | | | |

Kruskal-Wallis for continuous data, chi-squared tests ($\chi$2) for categorical data, and Bonferroni adjusted post hoc comparisons.

a (A-T-) b (A-T+) c (A+T-) d (A+T+) *p-value* < 0.05

e Repeat-measured follow-up cognition was predicted by a linear mixed model after controlling for age, sex, education, APOE, baseline cognition as a fixed variable, and follow-up time as a random variable

f After controlling for age, sex, education, APOE, linear model was used to predict baseline cognition

g Standardized regression coefficients represent correlations between different AT groups and cognition, the higher the value, the stronger the correlation between the group and the cognition

h after using predictive model control covariate estimates of cognition

i coefficient of determination, Indicates the degree to which the regression model interprets the predicted results

**Supple Table 5: Annual cognitive change rates using linear mixed model in different AT groups**

| **Predict ADAS_13 change rate (%)** | **A-T- (n=379)** | **A+T- (n=134)** | **A-T+ (n=48)** | **A+T+ (n=200)** | ***p* (>F)** |
|---|---|---|---|---|---|
| **follow-up 1 year** | 0.0965 | -0.00491 | 0.00734 | 0.0722 | 0.362893 |
| **follow-up 2 year** | 0.1232 | 0.1059 | 0.2609 | 0.1898 | 0.03255* |
| **follow-up 3 year** | 0.0706 | 0.2798 | -0.099 | 0.2135 | 0.0677173 |
| **follow-up 4 year** | 0.2395 | 0.1543 | 0.2768 | 0.2953 | 0.22807 |
| **Follow-up time (Years)** | 5.21 (2.8) | 5.10 (2.9) | 5.06 (2.7) | 4.12 (2.3) | |
| **Predict ADNI-MEM change rate (%)** | **A-T- (n=376)** | **A+T- (n=132)** | **A-T+ (n=48)** | **A+T+ (n=198)** | |
| **follow-up 1 year（median）** | 0.218 | 0.367 | 1.61 | 0.159 | 0.01566 * |
| **follow-up 2 year** | -0.642 | 0.017 | -0.146 | 1.068 | 0.5166 |
| **follow-up 3 year** | -0.201 | -0.373 | -0.098 | 0.285 | 0.2067 |
| **follow-up 4 year** | 0.287 | 1.415 | -10.34 | -0.229 | 0.0003915*** |
| **Follow-up times (Years)** | 4.97 (2.7) | 4.65 (2.5) | 4.95 (2.6) | 4.08 (2.3) | |
| **Predict MMSE change rate (%)** | **A-T- (n=383)** | **A+T- (n=134)** | **A-T+ (n=49)** | **A+T+ (n=199)** | |
| **follow-up 1 year** | -0.0014 | -0.0138 | -0.01027 | -0.0311 | 3.124e-05 *** |
| **follow-up 2 year** | 0.0031 | -0.0071 | -0.0133 | -0.0334 | 0.0001771 *** |
| **follow-up 3 year** | -0.0019 | -0.016 | 0.0225 | -0.0589 | 0.003001** |
| **follow-up 4 year** | -0.0006 | -0.0208 | -0.0172 | -0.0574 | 0.02762* |
| **Follow-up time (Years)** | 5.21 (2.9) | 5.10 (2.9) | 5.06 (2.7) | 4.12 (2.3) | |
| **Convert to dementia (rates)** | 40 (10.5%) | 32 (23.9%) | 8 (16.3%) | 84 (42%) | |
| **Dementia to normal (rates)** | 41 (10.6%) | 16 (11.9%) | 4 (8.2%) | 7 (3.5%) | |

MMSE: Mini-Mental State Examination; ADAS-13: 13-item Alzheimer's Disease Assessment Scale-cognitive subscale; ADNI_MEM: Alzheimer Disease Neuroimaging Initiative Memory Score. *Linear mixed prediction model controlled for sex and age education baseline cognitive estimates of the annual

cognitive change rate (List only the first four years). Kruskal-Wallis for continuous data, chi-squared tests (χ2) for categorical data, and Bonferroni adjusted post hoc comparisons. Aβ positivity (A+) was defined as FBP and FBB SUVR exceeding 1.11 and 1.08, respectively.; Tau positivity (T+) was defined as CSF p-Tau>26.64 pg/ml; Pr (>F): F test is used to analyze Linear mixed prediction modes that use more than one parameter to determine whether AT in the model is suitable for estimating the cognitive change rate (%).

**Supple Table 6: Stronger predictive value of Aβ pathology for cognitive decline in ApoE ε4 carriers compared to tau accumulation**

| **Groups (AT)** | **A-T+** | | **A+T-** | | *p*-value 1 | *p*-value 2 |
|---|---|---|---|---|---|---|
| APOE-ε4 classification | APOE-ε4+ | APOE-ε4- | APOE-ε4+ | APOE-ε4- | | |
| **Prediction of follow-up cognition** | | | | | | |
| **ADAS-13 fu** | 12.376 [c](N=41) | 8.652 [c](N=104) | 14.109 [a, b, d](N=245) | 11.847 [c](N=192) | 0.000 | 0.000 |
| **ADNI-MEM fu** | 0.423 (N=47) | 0.394 [c] (N=130) | 0.980 [b](N=302) | 0.638 (N=251) | 0.000 | 0.0032 |
| **MMSE fu** | 29.032 [b, d, c] (N=27) * | 28.094 [a] (N=68) | 28.346 [a, d](N=197) | 27.861 [a, c](N=148) | 0.000 | 0.0009 |

ADNI: Alzheimer's Disease Neuroimaging Initiative; APOE, apolipoprotein E; ADAS-Cog, Alzheimer Disease Assessment Scale-Cognitive; MMSE, Mini-Mental State Examination; ADNI_MEM: Alzheimer's Disease Neuroimaging Initiative Memory Score. *N: total number of repeat visits per year during follow-up. Data are shown as P50 or n (%). Kruskal-Wallis for continuous data, chi-squared tests (χ2) for categorical data, and Bonferroni adjusted post hoc comparisons. Aβ positivity (A+) was defined as FBP and FBB SUVR exceeding 1.11 and 1.08 on FBP and FBB PET, respectively; T+ defined by CSF p-Tau>26.64 pg/ml. *p*-value 1 was obtained by comparison of four groups, *p*-value 2 was obtained by comparison of each AT. a (A-T+ APOE-ε4+) b (A-T+ APOE-ε4-) c (A+T- APOE-ε4+) d (A+T+ APOE-ε4-) *p-value* < 0.05

**Supple Table 7: Baseline and follow-up cognitive status of different AT groups under different diagnoses.**

| **Group AT** | **A-T-** | **A+T-** | **A-T+** | **A+T+** | ***p*** |
|---|---|---|---|---|---|
| *** Baseline cognition of CN** | **N=152** | **N=36** | **N=12** | **N=35** | **<0.001** |
| **MMSE ba** | 29.0± 0.6 [b,c,d] | 27.8± 0.5 [a,c,d] | 28.5± 0.5 [a,b,d] | 27.2± 0.5 [a,b,c] | **<0.001** |
| **ADAS-13 ba** | 8.9± 2.1 [b,c,d] | 14.5± 2.1 [a,c,d] | 11.3± 1.7 [a,d] | 17.0± 2.1[a,b,c] | **<0.001** |
| **ADNI-MEM ba** | 0.99± 0.26 [b,c,d] | 0.45± 0.26 [a,c,d] | 0.75± 0.2 [a,b,d] | 0.21± 0.25[a,b,c] | **<0.001** |
| **Baseline cognition of SMC** | **N=28** | **N=18** | **N=4** | **N=10** | **<0.001** |
| **MMSE ba** | 28.9± 0.6 [b,c,d] | 28.0± 0.5 [a,d] | 28.0± 0.6 [a,d] | 27.3± 0.5[a,b,c] | **<0.001** |
| **ADAS-13 ba** | 9.0± 2.1 [b,c,d] | 13.5± 2.0 [a,d] | 13.6±1.7 [a,d] | 16.6± 1.7[a,b,c] | **<0.001** |
| **ADNI-MEM ba** | 0.97± 0.26 [b,c,d] | 0.57± 0.25 [a,d] | 0.47± 0.20 [a] | 0.26± 0.2[a,b] | **<0.001** |
| **Baseline cognition of MCI** | **N=121** | **N=69** | **N=6** | **N=122** | **<0.001** |
| **MMSE ba** | 28.8± 0.6 [c,d] | 27.7± 0.5 [c,d] | 28.6± 0.6[a,b,d] | 27.1± 0.6 [a,b,c] | **<0.001** |
| **ADAS-13 ba** | 9.6± 2.0 [b,c,d] | 14.7± 1.9 [a,c,d] | 11.6±2.3 [a,b,d] | 17.4± 2.2 [a,b,c] | **<0.001** |
| **ADNI-MEM ba** | 0.90± 0.30 [b,c,d] | 0.42± 0.24 [a,c,d] | 0.72± 0.29 [a,b,d] | 0.16± 0.27 [a,b,c] | **<0.001** |
| **Baseline cognition of AD** | **N=3** | **N=5** | **N=4** | **N=29** | **<0.001** |
| **MMSE ba** | 28.4± 0.3 [d] | 27.7± 0.9 [d] | 28.0± 0.5 [d] | 26.9± 0.4 [a,b,c] | **<0.001** |
| **ADAS-13 ba** | 11.8± 0.9 [c,d] | 15.3± 3.0 [d] | 13.0± 2.2[a,d] | 18.2± 1.5[a,b,c] | **<0.001** |
| **ADNI-MEM ba** | 0.63± 0.10 [d] | 0.36± 0.38 [d] | 0.53± 0.25 [d] | 0.05± 0.19 [a,b,c] | **<0.001** |
| **Follow up cognition of CN** | **N=152** | **N=36** | **N=12** | **N=35** | **<0.001** |
| **MMSE fu** | 28.8±0.6 [b,c,d] | 26.0± 0.6 [a,c,d] | 27.5±0.5 [a,b,d] | 24.6±0.6[a,b,c] | **<0.001** |
| **ADAS-13 fu** | 9.5±2.3 [b,c,d] | 18.8± 2.3 [a,c,d] | 13.8±1.8 [a,b,d] | 23.1±2.2[a,b,c] | **<0.001** |
| **ADNI-MEM fu** | 1.04±0.34 [b,c,d] | 0.19± 0.33 [a,c,d] | 0.66±0.26 [a,b,d] | -0.19±0.33[a,b,c] | **<0.001** |
| **Follow up cognition of SMC** | **N=28** | **N=18** | **N=4** | **N=10** | **<0.001** |

| | | | | | |
|---|---|---|---|---|---|
| **MMSE fu** | 28.8± 0.7 [b,c,d] | 26.2± 0.6 [a,d] | 26.9± 0.7 [a,d] | 24.7± 0.5 [a,b,c] | **<0.001** |
| **ADAS-13 fu** | 9.6± 2.5 [b,c,d] | 17.8± 2.1 [a,d] | 16.5± 1.7 [a,d] | 22.8± 1.8 [a,b,c] | **<0.001** |
| **ADNI-MEM fu** | 1.0± 0.35 [b,c,d] | 0.34± 0.32 [a,d] | 0.31± 0.28 [a,d] | -0.13± 0.28 [a,b,c] | **<0.001** |
| **Follow up cognition of MCI** | **N=121** | **N=69** | **N=6** | **N=122** | **<0.001** |
| **MMSE fu** | 28.6± 0.7 [b,c,d] | 26.0± 0.6 [a,c,d] | 27.5± 0.7 [a,b,d] | 24.5± 0.7 [a,b,c] | **<0.001** |
| **ADAS-13 fu** | 10.3± 2.2 [b,c,d] | 19.2± 2.2 [a,c,d] | 14.0±2.8 [a,b,d] | 23.7± 2.4 [a,b,c] | **<0.001** |
| **ADNI-MEM fu** | 0.93± 0.33 [b,c,d] | 0.15± 0.31 [a,c,d] | 0.63± 0.47 [a,b,d] | -0.26± 0.36 [a,b,c] | **<0.001** |
| **Follow up cognition of AD** | **N=3** | **N=5** | **N=4** | **N=29** | **<0.001** |
| **MMSE fu** | 28.1± 0.3 [b,c,d] | 25.7± 1.0 [a,c,d] | 28.6± 0.6 [a,b,d] | 24.3± 0.5[a,b,c] | **<0.001** |
| **ADAS-13 fu** | 12.3± 0.8 [b,c,d] | 19.8± 3.3 [a,c,d] | 11.6±2.3 [a,b,d] | 14.7± 1.7 [a,b,c] | **<0.001** |
| **ADNI-MEM fu** | 0.59± 0.10 [c,d] | 0.07± 0.50 [d] | 0.72± 0.29 [a,d] | -0.41± 0.24 [a,b,c] | **<0.001** |

MMSE: Mini-Mental State Examination; ADAS-13: 13-item Alzheimer's Disease Assessment Scale-cognitive subscale; ADNI_MEM: Alzheimer Disease Neuroimaging Initiative Memory Score. *Linear models controlling for sex, age, education, APOE estimated baseline and follow-up cognition. Continuous data were tested by ANOVA, and post-hoc comparisons were performed by LSD. Aβ positivity (A+) was defined as FBP and FBB SUVR exceeding 1.11 and 1.08, respectively.; Tau positivity (T+) was defined as CSF p-Tau>26.64 pg/ml; *p* values are given overall among AT groups. a (A-T-) b (A+T-) c (A-T+) d (A+T+) *p-value* < 0.05
