# Supplementary material for: Amyloid‐β but not tau accumulation is strongly associated with longitudinal cognitive decline
Source: CNS Neurosci Ther. 2024 Jul 16;30(7):e14860. doi: 10.1111/cns.14860 (PMC11251873; doi:10.1111/cns.14860)
Supplement: Supplementary file 1 — Data S1 [file CNS-30-e14860-s001.zip › cns14860-sup-0001-SupinfoS1/Figure Legends.docx]

**Supple Fig. 1** **The trajectory of longitudinal memory performances** (**a**) Mean ADAS-13 at each year of follow-up in different AT groups; (**b**) Mean ADNI-MEM at each year of follow-up in different AT groups;(**c**) Mean MMSE at each year of follow-up in different AT groups; Dot sizes are proportional to the number of observations;(**d**) Sankey diagrams depict AT classification across diagnostic status and APOE; (**e**) Sankey diagrams depict AT classification across diagnostic status and cognitive change. Concrete temporal trajectories of memory tests in each AT group and the corresponding numbers are shown in **Supple Table 3.**

**Supple Fig. 2** **The relationship between the AT biomarkers and cognition performances**. Follow-up cognition was estimated by a linear mixed model (controlling for age, sex, education, APOE, baseline cognition as a fixed factor, and follow-up duration as a random factor);(**a**) regression fitting of CSF tau and model-estimated ADAS-13 scores at the Aβ level;(**b**) regression fitting of CSF tau and model-estimated MMSE scores at the Aβ level; (**c**) regression fitting of CSF tau and model-estimated ADNI-MEM scores at the Aβ level; (**d**) regression fitting of amyloid-PET and model-estimated ADAS-13 scores at the tau level; (**e**) regression fitting of amyloid-PET and model-estimated MMSE scores at the tau level; (**f**) regression fitting of amyloid-PET and model-estimated ADNI-MEM scores at the tau level;(**g**)Scatterplot showing the correlations between CSF-Aβ and amyloid-PET levels in all participants; (**h**)Scatterplot showing the correlations between amyloid-PET and CSF-tau levels in all participants. scatter plots red for Aβ+, blue for Aβ-(**a-c**); scatter plots red for tau+, blue for tau-(**d-f**). standardized β- and p-values were derived from linear regression. The number of follow-up cognitions was superimposed on the number of repeated measures and the number of follow-up visits. R 2 represents the degree of fit of the mixed-effects model. Cor is the correlation coefficient (Pearson). p≤0.05 was statistically significant. Concrete model estimates are shown in **Supple Table 7.**

**Supple Fig. 3 Predictive value of A and T for cognitive decline in CD33 risk allele carriers.** Violin plots illustrate the distribution of baseline and following-up cognition scores including ADAS-13 (**a, b**), ADNI-MEM (**c, d**), and MMSE (**e, f**) estimated after the linear mixed model (controlling for age, sex, education, baseline cognition as a fixed factor and controlling for follow-up time as a random factor) across the different AT groups (A+T- and A-T+) and CD33 (AA vs CC) groups; The central black lines show the median value, upper and lower quartiles, respectively display the upper and lower quartiles of the black line; the white diamond represents the mean of cognition; the red points are outliers. **p* ≤ 0.01, ***p* ≤ 0.001. ****p* ≤ 0.0001.

**Supple Fig. 4 The patterns and region-specific effects of Aβ (A+T-) and tau pathology (A-T+) on baseline FDG-PET SUVR and longitudinal change.** A voxel two-sample t-test was performed for FDG-PET in A-T + and A+T- groups (**a, c**); The levels of 2-year-follow-up FDG-PET change in A-T+ and A+T- groups (**b, d, e**); after FWE corrected *p* ≤ 0.05 meaningful brain regions show as above said two samples in FDG level difference was statistically significant. The central black lines show the median value, upper and lower quartiles, respectively display the upper and lower quartiles of the black line; the white diamond represents the mean of cognition; the red points are outliers; the weight of the violin represents the density of the population distribution.
